# A multiscale mathematical model of cell dynamics during neurogenesis in the mouse cerebral cortex

**DOI:** 10.1186/s12859-019-3018-8

**Published:** 2019-09-14

**Authors:** Marie Postel, Alice Karam, Guillaume Pézeron, Sylvie Schneider-Maunoury, Frédérique Clément

**Affiliations:** 1 Sorbonne Université, Université Paris-Diderot SPC, CNRS, Laboratoire Jacques-Louis Lions, LJLL, Paris, France; 2 Sorbonne Université, CNRS UMR7622, Inserm U1156, Institut de Biologie Paris-Seine (IBPS), Laboratoire de Biologie du développement (LBD), Paris, France; 3 Inria, Université Paris-Saclay, Palaiseau, France; 4 LMS, Ecole Polytechnique, CNRS, Université Paris-Saclay, Palaiseau, France; 5 Current address: Laboratoire Physiologie Moléculaire et Adaptation, UMR 7221 CNRS, Muséum National d’Histoire Naturelle, Paris, France

**Keywords:** Multiscale mathematical modeling, Neurogenesis, Development of the cerebral cortex, Cell dynamics, Neural progenitors, Mouse mutant for *F*tm/Rpgrip1l, Numerical simulations, Time varying transfer rates, Cell cycle indexes

## Abstract

**Background:**

Neurogenesis in the murine cerebral cortex involves the coordinated divisions of two main types of progenitor cells, whose numbers, division modes and cell cycle durations set up the final neuronal output. To understand the respective roles of these factors in the neurogenesis process, we combine experimental in vivo studies with mathematical modeling and numerical simulations of the dynamics of neural progenitor cells. A special focus is put on the population of intermediate progenitors (IPs), a transit amplifying progenitor type critically involved in the size of the final neuron pool.

**Results:**

A multiscale formalism describing IP dynamics allows one to track the progression of cells along the subsequent phases of the cell cycle, as well as the temporal evolution of the different cell numbers. Our model takes into account the dividing apical progenitors (AP) engaged into neurogenesis, both neurogenic and proliferative IPs, and the newborn neurons. The transfer rates from one population to another are subject to the mode of division (proliferative, or neurogenic) and may be time-varying. The model outputs are successfully fitted to experimental cell numbers from mouse embryos at different stages of cortical development, taking into account IPs and neurons, in order to adjust the numerical parameters. We provide additional information on cell kinetics, such as the mitotic and S phase indexes, and neurogenic fraction.

**Conclusions:**

Applying the model to a mouse mutant for *Ftm/Rpgrip1l*, a gene involved in human ciliopathies with severe brain abnormalities, reveals a shortening of the neurogenic period associated with an increased influx of newborn IPs from apical progenitors at mid-neurogenesis. Our model can be used to study other mouse mutants with cortical neurogenesis defects and can be adapted to study the importance of progenitor dynamics in cortical evolution and human diseases.

**Electronic supplementary material:**

The online version of this article (10.1186/s12859-019-3018-8) contains supplementary material, which is available to authorized users.

## Background

The multiple functions of the mammalian cerebral cortex in integrating sensory stimuli, controlling motor output and mediating cognitive functions are supported by an extraordinary diversity of neuronal subtypes mutually connected through complex neuronal circuitry. The formation of this structure requires producing the correct numbers and subtypes of neurons at the proper position during a specific period of embryonic and fetal development, the neurogenesis period, which lasts several days in mice to several months in humans [1–3].

The cerebral cortex is a laminar structure in the dorsal telenceph/alon, composed of two major classes of neurons, pyramidal neurons and interneurons. We focus on pyramidal neurons, which are more abundant and, unlike interneurons, are generated by local progenitors in the dorsal telencephalon. Apical progenitors (APs) in the cortical ventricular zone (VZ) give birth to populations of basal progenitors (BPs) forming the subventricular zone (SVZ), mainly populated in the mouse cortex with intermediate progenitors (IPs)) [4, 5].

**Fig. 1:**
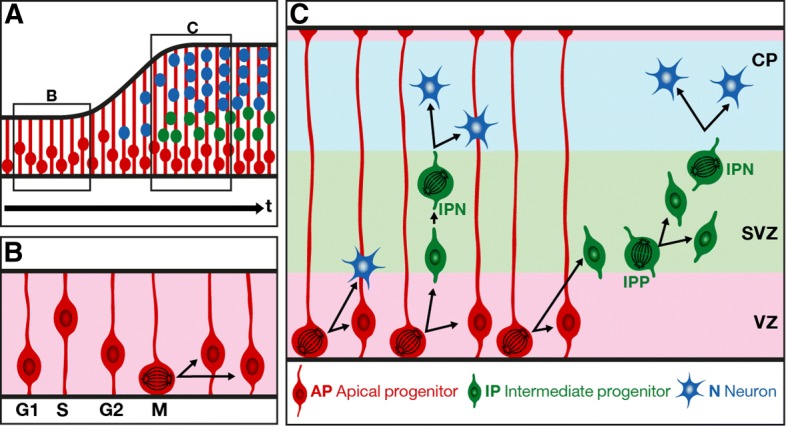
Schematic representation of cortical neurogenesis during mouse brain development. Dividing apical progenitor nuclei undergo interkinetic nuclear migration (INM): their nuclei move within the ventricular zone, from the apical surface toward basal (G1-S phases) then back to the apical surface (S-G2 phases) where they divide (M phase). **a** Representation of the cortex at different developmental stages. The black arrow represents developmental time. Boxed areas **b** and **c** correspond to stages before neurogenesis and during neurogenesis, respectively. **b** Before the onset of neurogenesis, APs divide symmetrically to give rise to two new APs, leading to an expansion of the AP population and consequently of the ventricular surface. **c** From the onset of neurogenesis (E12.5) until the end of gestation (E18.5), APs divide asymmetrically to self-renew and to give rise to a neuron that migrates toward the cortical plate (CP) (direct neurogenesis) or to an IP (IP-genic division) that migrates out of the ventricular zone (VZ) to form the subventricular zone (SVZ). IPs can either be IPNs that divide to give rise to 2 neurons or IPPs that divide to give rise to two IPNs

Cortical progenitors display distinct modes of division (illustrated in Fig. 1 for the mouse cortex) [1] and cell cycle parameters [6]. Before the onset of neurogenesis in mouse embryos, APs divide symmetrically to expand the progenitor pool. At the onset of neurogenesis, APs start to divide asymmetrically to self-renew and produce either one neuron (direct neurogenesis) or one IP that migrates into the SVZ. IPs divide symmetrically. Neurogenic IPs (IPNs) give birth to two neurons directly after one cell cycle. Proliferative IPs (IPPs) give birth to two neurogenic IPs, each of which will in turn give rise to two neurons. Young neurons then migrate radially toward the cortical plate [4, 5, 7–9]. IPs are produced throughout cortical neurogenesis, thereby contributing to all cortical layers [9].

Many factors contribute to the dynamics of neurogenesis and to the final neuronal number: the timing of initiation and termination of neurogenesis, the initial number of apical progenitors, the ratio of each division type, the duration of the cell cycles. Moreover, in pathological contexts, several factors may be modified simultaneously, complicating the interpretation. The analysis of these pathological situations would thus highly benefit from modeling approaches taking into account the dynamics of the different cell types.

So far, modeling approaches of cell lineage dynamics in the developing cortex have mainly used the branching process formalism. (see e.g. [10–13]). These approaches generally focus on the impact of division types (self-amplyfing, self-renewing or self-consumming, following the terminology proposed in [14]). Apoptosis can also be considered in developmental toxicology studies [13, 15]. Such approaches can lead to interesting theoretical results on the properties of the population growth regime [10] or the cell lineage trees (size and degree of imbalance) [12]. Most of the time, the notion of progenitor cell is quite generic, except in some cases where model cell types can be mapped to biological cell types [11]. When it exists, the confrontation of the model outputs to experimental data is carried out on the whole population. This is due to the fact that the natural time increment in these models is expressed as the cell generation number. To relate this number to a time unit expressed as embryonic days, one needs to apply simplifying assumptions on the cell cycle duration considered as constant over all cell types and/or transit times all along the cell cycle (stationary uniform distribution of cells within the cell cycle). Interestingly, a recent work focused on IP dynamics to explain compensation for induced neuronal death in the mouse cortex [16]. Through stochastic simulations based on the Gillespie algorithm, the authors investigated the possible impact of several IPgenic divisions, and alteration in IP cell cycle duration.

The importance of distinguishing the different types of cells which derive from one another has also been established in other contexts (see for instance [17] for the germinative compartment of the epidermis and [18] for the cerebellum).

We also mention here some recent works dealing with cell dynamics in adult hippocampal neurogenesis, even if it differs from developmental neurogenesis, especially as for the involvement of apoptosis in non pathological situations. In particular, in [19], the authors introduced a multitype Bellman-Harris branching process, which explicitly takes into account the cell cycle phases and generation number of amplifying neuroprogenitors. They took advantage of the stationary nature of adult neurogenesis on the short term to obtain experimentally plausible cell numbers (from raw data expressed as proportion of BrdU labeled cell types). In contrast, the motivation of [20, 21] was mainly to explain the long term decay in the hippocampal pool of neural stem cells, by means of a deterministic compartmental model and its stochastic counterpart with similar average transit times (hence cell cycle durations) within each compartment.

Finally, a series of studies [22–25]) have investigated embryonic cortical neurogenesis on its whole time horizon, with an evolutionary perspective. These studies compare the management of cell resources between species through two main factors: the quit fraction (equivalent to a cell cycle exit rate), and the time-varying doubling times of proliferative cells. They focus on the global neuronal output obtained from an inferred precursor pool size. Yet, they cannot provide accurate information on cell kinetics, since they do not take into account the lineage structure of the different cell populations within the cortex. The cumulative number of neurons generated during neurogenesis is not only determined by the initial number of neural progenitors (sometimes called founder population in the literature) and the duration of their proliferative, neurogenic (versus gliogenic) period, but also by their lineage [26]. Importantly, neurogenic lineages involve heterogenous populations of progenitors, including transit amplifying cells such as IP, in addition to self-renewing cells and post-mitotic cells. Hence, as analyzed in a rigorous and thorough mathematical way in [17], equations for homogeneous cell populations cannot be applied to hierarchical cell populations as encountered during neurogenesis, as well as many other physiological processes based on cell dynamics such as epidermis renewal [17], hematopoiesis [27], or renewal of intestinal crypts [28]. In particular, on the population level, the mixing of asymmetric and symmetric neurogenic divisions results in a combination of linear and exponentiel increase in the number of generated neurons. Also, the hierarchical structure of the lineage impacts the distribution of each cell type along the cell cycle (age distribution) and all cell kinetics parameters deriving from this distribution, such as the cell cycle duration, growth or quit fraction, and proliferation (labeling and mitotic) indexes. As a consequence, parameters from self-renewing and transit amplifying cells should not be lumped together.

The current study was motivated by the observation of complex cortical neurogenesis defects in a mouse knock-out mutant for the *Ftm/Rpgrip1l* gene (hereafter called the *Ftm* mutant). The *Ftm/Rpgrip1l* gene encodes a protein involved in the formation and function of the primary cilium [29–31], a sensory organelle projecting from the cell surface with multiple functions in development and whose defects cause human syndromes called ciliopathies [32, 33]. At the peak of cortical neurogenesis (around embryonic stage E14.5), *Ftm*^−/−^ fetuses show a strong reduction in the numbers of neurons and IPs. Intriguingly, at later stages the neurogenesis defect is at least partly compensated for.

In this work, we explore further whether this compensation may be due to differences in the neurogenic potential of APs and IPs, and/or in the proliferation potential of IPs, and especially in the proportion of IPs undergoing two consecutive cell cycles (IPgenic IPs, IPPs) instead of one (neurogenic IPs, IPNs). Additionally, we set up a framework in which one can study the impact of the durations of the whole cell cycle and of any of the cell cycle phases. We aim to quantify the impact on the final neuronal output of changes in progenitor proliferation/neurogenesis rates at different developmental stages, to interpret our mutant phenotypes and possibly to guide future experiments.

We combine an experimental quantitative in vivo investigation of the different cell types along the whole neurogenesis process with the design of a mathematical model for cell population dynamics, which is derived from cell-kinetics principles and based on a deterministic formalism. Numerical simulations of our model allows one to follow the numbers of (i) dividing APs committed to neurogenesis, (ii) newborn neurons, and (iii) neurogenic and IPgenic IPs. The transfer rates from one population to another are subject to the type of division and may be time-varying.

The simulated cell numbers are fitted to the experimental numbers to calibrate the parameters entering the formulations of the transfer rates. In the case of (either neurogenic or IPgenic) IPs, one can also follow the progression along the subsequent phases of the cell cycle (G1, S, G2, M). Additional biological outputs related to IPs are thus available, such as the mitotic index (MI) and labeling index (LI, also called S-phase index), as well as MI- or LI- derived information (e.g. the fraction of neurogenic mitoses). The computation of such outputs also enables us to illustrate how the model can be used to supply computer-assisted help in the design and assessment of experimental protocols devoted to cell kinetics analysis.

We describe the quantitative changes in the AP, IP and neuron populations during the whole neurogenesis period in mice. From information on global cell numbers, we can derive information on the rate and mode of cell divisions underlying the neural lineage, and track in an accurate way the progression of IPs along the cell cycle. Comparisons of the results obtained from the wild-type and *Ftm* mutant mice suggest that a major effect of the *Ftm* mutation is to shorten the duration of the neurogenic period, which appears to start later, while it ends up at a similar time with an only slightly reduced neuronal yield. Together with the neurogenesis shortening, the compensation for neuron production requires an intensive recruitment of committed APs at mid-neurogenesis, where the IP numbers exhibit a narrow high-amplitude peak. Our modeling approach, based on data-driven outputs, allows us to monitor the time course of IP progenitors and neurogenic AP inflow in both control and mutant situations. All symbols and notations are summarized in Table 1.

**Table 1:** Notations used for variables and parameters in the model formulation

Notation	Unit	Definition	Description
AP		Apical progenitor	
IP		Intermediate progenitor	
N		Neuron	
IPN		Neurogenic IP	IP that divides symmetrically to give two Ns
IPP		Proliferative / IPgenic IP	IP that divides symmetrically to give rise to two IPNs
*F*_*AP*_(*t*)	cell nb/hour	AP inflow into neurogenesis	Instantaneous AP Number fuelling neurogenesis
*K*_*AP*_	cell nb/hour	Parameters of *F*_*AP*_(*t*) formulated in (Eq. (16))	Scaling factor
*t*_+_	day		Location of the rise inflexion point
*s*_+_	day ^−1^		The slope of *F*_*AP*_(*t*) at *t*^+^ is approximately 4*s*^+^*K*_*AP*_
*t*_−_	day		Location of the decay inflexion point
*s*_−_	day ^−1^		The slope of *F*_*AP*_(*t*) at *t*^−^ is approximately −4*s*^−^*K*_*AP*_
*β*(*t*)		Ratio of IP-genic divisions of APs	Proportion of fated AP subject to direct neurogenesis
*γ*(*t*)		Ratio of IPP-genic AP divisions	Proportion of fated AP subject to an IPgenic division entering an IPP cell cycle
*γ*_0_		Parameters of *γ*(*t*) formulated in a sigmoid (Eq. (15))	Limit value of *γ*(*t*) for *t*→−*∞*
*γ*_1_			Limit value of *γ*(*t*) for *t*→+*∞*
*t*_*γ*_	day		Location of inflexion point of *γ*(*t*)
*s*_*γ*_	day ^−1^		4*s*_*γ*_/(*γ*_1_−*γ*_0_) is the slope of *γ*(*t*) at *t*_*γ*_
*IPP*(*t*,*a*)	cell nb/hour	Density of cells at time *t* and age *a*	Density of cells of type IPP, solution of PDE (Eq. (1))
*IPN*(*t*,*a*)	cell nb/hour	Density of cells at time *t* and age *a*	Density of cells of type IPN, solution of PDE (Eq. (2))
$\overline {IPP} (t)$	cell nb	Number of cells of type IPP	Number of cells IPP at time *t*.
$\overline {IPN} (t)$	cell nb	Number of cells of type IPN	Number of cells IPN at time *t*.
*a*	hour	age	Location within the cell cycle
$\overline {X}_{P} (t)$	cell number	X in the *P* phase	X∈{IPP,IPN,IP}; *P*∈{*G*1,*S*,*G*2,*M*}
*LI*(*t*)		Labeling index (Eq. (13))	Proportion of cells in *S* phase over the total number of cycling cells (defined for a specific progenitor type)
*MI*(*t*)		Mitotic index (Eq. (14))	Proportion of cells in M phase over the total number of cycling cells (defined for a specific progenitor type)
*ψ*(*t*)		IP neurogenic fraction (Eq. 12)	Proportion of IP mitoses fated to neurogenic divisions
*ρ*(*t*,*Δt*)		Proportion of IP cells in S phase at *t* and *t*+*Δt* detected by double labeling (Eq. (25))	Efficiency of detection of cells undergoing a second S phase by double-labeling techniques based on a large delay *Δt*
$r^{N}_{AP}$		Neuronal yield (Eq. (19))	Ratio of the final number of produced neurons over the cumulated number of AP that have entered neurogenesis

## Results

### Design of the mathematical model and model outputs

The model investigates the neurogenesis steps occurring from the initiation of AP engagement in neurogenesis (hence the apparition of neurogenic or IPgenic divisions of APs) until neurogenesis ending (which corresponds to a switch to gliogenesis in normal conditions [34, 35]).

#### General framework

The general structure of the model is based on a deterministic compartmental model with 4 compartments : an inflow compartment corresponding to APs engaged in neurogenesis, an outflow compartment corresponding to neurons, and in between, the compartments corresponding to proliferative and neurogenic IPs.

**Fig. 2:**
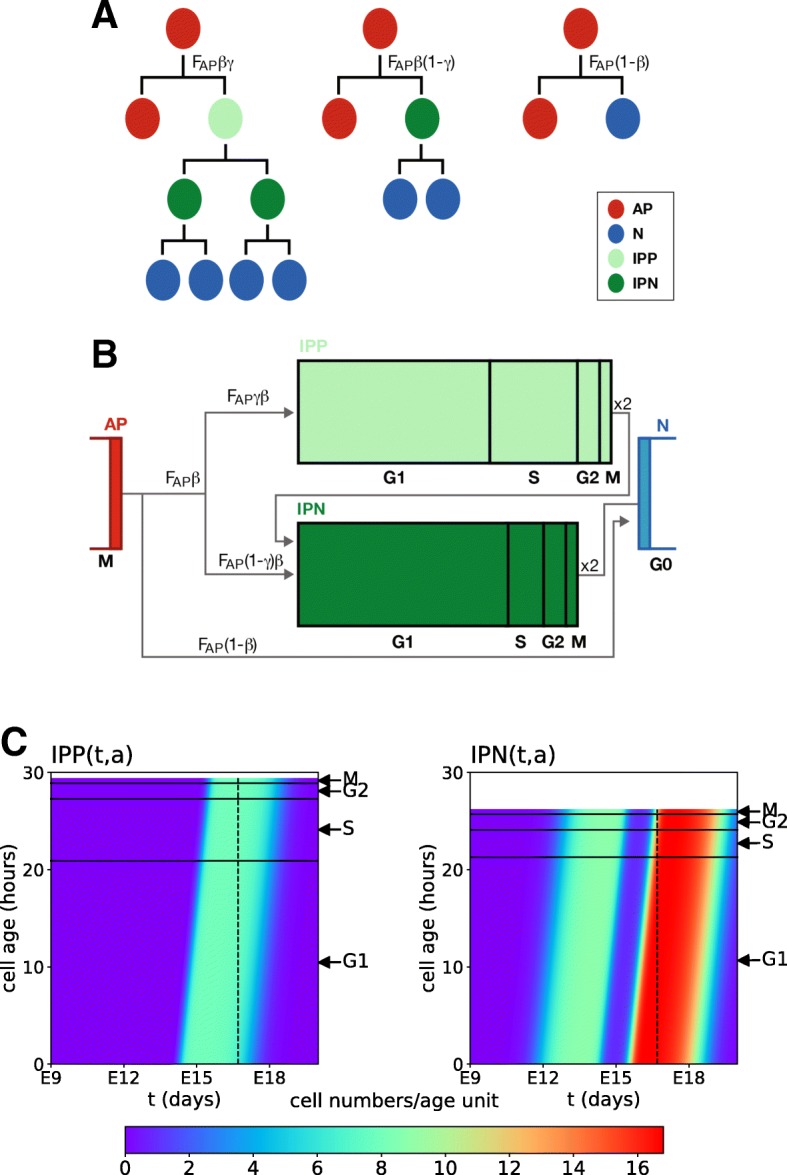
Modes of division of cortical progenitors and cell cycle phases. **a** Schematic diagram of the different modes of neural progenitor cell divisions **b** Another representation of progenitor divisions highlighting the duration of the cell cycle phases according to [6]. Note the difference in the duration of phases G1 and S in IPPs as compared to IPNs (see Table 3). **c** Display of the IPP and IPN cell densities in the age/time plane, computed using the model, for the parameter set 3 from Table 4. The same color code is used for both IPPs and IPNs (number of cells per age unit). The horizontal axis corresponds to the time, and the vertical axis to the age of the cells within the cell cycle. The horizontal black lines indicates the G1, S, G2, and M phases
- the production of IPgenic IPs (IPs that will complete two successive cell cycles and be at the source of IPNs at the end of their first cycle) with rate *β*(*t*)*γ*(*t*) (transition *AP*→*IPP*).

The instantaneous inflow of APs, denoted by *F*_*AP*_(*t*) (Fig. 2) is distributed amongst three cell types according to possibly time-varying rates. APs engaged in neurogenesis can either undergo direct neurogenesis (transition *AP*→*N*) with rate (1- *β*(*t*)) or give birth to intermediate progenitors with rate *β*(*t*). The non neurogenic AP flow is further separated into:

- the production of neurogenic IPs (IPs that will complete a single cell cycle before engendering neurons through transition *IPN*→*N*) with rate *β*(*t*)(1−*γ*(*t*)) (transition *AP*→*IPN*);

Hence, there are two incoming flows in the IPN compartment : one stemming from APs and another from IPPs. Embedded within this compartment structure, the dynamics of IP cell populations are described in more details and encompass several scales.

#### Microscopic scale

The model describes the progression along the cell cycle and the repartition into the different cell cycle phases of the intermediate progenitors through their local cell densities *IPP*(*t*,*a*) and *IPN*(*t*,*a*) (number of cells per hour). The first variable *t* denotes the time, measured in embryonic days, and the second variable *a* is the cytological age (i.e. the time elapsed since last mitosis), measured in hour.

The evolution of the cell densities *IPP*(*t*,*a*) and *IPN*(*t*,*a*) is the outcome of a linear non conservative transport equation with unit speed

1 $$\begin{array}{@{}rcl@{}}  \left\{\begin{array}{l}\partial_{t} IPP(t,a)+\partial_{a} IPP(t,a)=0,\quad t>t_{0}, ~a\in ]0,T_{C}^{IPP}[\\ IPP(t_{0},a)=IPP_{0}(a),\end{array}\right. \end{array} $$

2 $$\begin{array}{@{}rcl@{}} \left\{\begin{array}{l}\partial_{t} IPN(t,a)\,+\,\partial_{a} IPN(t,a)\,=\,0,\quad t>t_{0}, ~a\in ]0,T_{C}^{IPN}[\\ IPN(t_{0},a)=IPN_{0}(a),\end{array}\right. \end{array} $$

where $T_{C}^{IPP}$ and $T_{C}^{IPN}$ are the cell cycle durations of respectively IPgenic and neurogenic IPs, which set the (constant) length of the numerical domains (as seen in Fig. 2, this domain is longer for IPPs, since $T_{C}^{IPP}>T_{C}^{IPN}$).

The initial conditions *IPP*_0_(*a*) and *IPN*_0_(*a*) will be set to zero in practice, since the initial time *t*_0_ corresponds to the start of neurogenesis from APs, before which no IP has yet been produced.

Systems (1) and (2) are accompanied by proper boundary conditions (3-5) representing the coupling between the different cell types: AP inflow into IPP or IPN, IPP inflow into IPN, and IPN outflow into N.

New born IPPs entering the IPP domain through the left boundary, at *IPP*(*t*,*a*=0), come integrally from AP divisions.

3 $$\begin{array}{@{}rcl@{}} IPP(t,a=0)&=&\gamma(t)\beta(t)F_{AP}(t). \end{array} $$

Once they have reached the right boundary, at $IPP(t,a=T_{C}^{IPP})$, IPPs divide and the resulting IPP flow is entirely and immediately transported onto the left boundary of the IPN domain (note factor 2 to account for mitosis). New born IPN cells, at *IPN*(*t*,*a*=0), can arise either from AP or former IPP divisions.

4 $$ {}{\begin{aligned} IPN(t,a=0)=(1-\gamma(t))\beta(t)F_{AP}(t)+2IPP\left(t,a=T_{C}^{IPP}\right). \end{aligned}}  $$

Once they have reached the right boundary of the IPN domain, at $IPP(t,a=T_{C}^{IPN})$, cells become newborn post-mitotic neurons. The rate of neuron birth is further increased by the contribution of direct neurogenesis from APs:

5 $$\begin{array}{@{}rcl@{}} \frac{dN}{dt}(t)&=&(1-\beta({t}))F_{AP}(t)+2IPN\left(t,a=T_{C}^{IPN}\right). \end{array} $$

While the total lengths of the age domains correspond to the cell cycle durations, the locations of their internal boundaries correspond to the durations of the different cell cycle phases, which differ between neurogenic and IPgenic IPs:

$$\begin{array}{@{}rcl@{}} T_{C}^{IPP}&=&T_{G1}^{IPP}+T_{S}^{IPP}+T_{G2}^{IPP}+T_{M}^{IPP},\\ T_{C}^{IPN}&=&T_{G1}^{IPN}+T_{S}^{IPN}+T_{G2}^{IPN}+T_{M}^{IPN}. \end{array} $$

With specific choices of the *β*(*t*), *γ*(*t*) and *F*_*AP*_(*t*) functions, Eqs. (1-4) can be solved numerically using the method of characteristics (see Methods). The 2D visualization in Fig. 2c enables one to follow the changes in the distributions of IPPs (left panel) and IPNs (right panel). The horizontal axis corresponds to time, and the vertical axis to the cell age. The horizontal black lines mark the locations of inner boundaries delimiting the cell cycle (G1,S, G2 and M) phases, each having different durations in IPPs and IPNs. The same color code is used for both IPPs and IPNs. Neurogenesis starts a little before E12 and results in the appearance of newborn cells in both the IPP and IPN domains. At E17, the inflow from APs dries up and at E18, the last IPPs complete mitosis and enter the IPN compartment which, in turn, empties around E19.

#### Macroscopic scale

For any cell type IP, IPP, IPN or N, we can compute the cell number as a function of time.

For IPPs and IPNs we can compute the number of cells at a given time by integrating the age distribution over the whole cell cycle duration $\left (T_{C}^{IPP}\ \text {or}\ T_{C}^{IPN}\right)$

6 $$\begin{array}{@{}rcl@{}} \overline{IPP}(t)&=&\int_{0}^{T_{C}^{IPP}}IPP(t,a)da, \\  \overline{IPN}(t)&=&\int_{0}^{T_{C}^{IPN}}IPN(t,a)da, \end{array} $$

from which we can compute the total number of IP cells

7 $$\begin{array}{@{}rcl@{}} \overline{IP}(t)&=&\overline{IPP}(t)+\overline{IPN}(t). \end{array} $$

The number of neurons at a given time N(t) is obtained directly from integrating (5) in time

8 $$\begin{array}{@{}rcl@{}} N(t)&=&\int_{0}^{t}\left((1-\beta(\tau))F_{AP}(\tau)+2IPN\left(\tau,T_{C}^{IPN}\right)\right)d\tau. \end{array} $$

#### Mesoscopic scale

In the case of IPPs and IPNs, we can compute partial cell numbers within each phase of the cell cycle, and especially phases S and M, so that we can also derive expressions for the mitotic (MI; ratio of cells in M phase) and labeling (LI; ratio of cells in S phase) indexes in the IP population. We obtain

9 $$\begin{array}{@{}rcl@{}} \overline{IPP}_{S}(t)&=&\int_{T_{G1}^{IPP}}^{T_{G1}^{IPP}+T_{S}^{IPP}}{IPP}(t,a)da, \end{array} $$

10 $$\begin{array}{@{}rcl@{}}  {\overline{{IPP}}_{M}(t)}&=&{\int_{T_{c}^v-T_{M}^{IPP}}^{T_{c}^{IPP}}{IPP}(t,a)da,}\end{array} $$

11 $$\begin{array}{@{}rcl@{}} \overline{IPN}_{S}(t)&=&\int_{T_{G1}^{IPN}}^{T_{G1}^{IPN}+T_{S}^{IPN}}{IPN}(t,a)da, \end{array} $$

12 $$\begin{array}{@{}rcl@{}}  {\overline{{IPN}}_{M}(t)}&=&{\int_{T_{c}^{IPN}-T_{M}^{IPN}}^{T_{c}^{IPN}}{IPN}(t,a)da,}\end{array} $$

from which we drive the expressions for the labeling and mitotic indexes

13 $$\begin{array}{@{}rcl@{}}  LI(t)&=&\frac{\overline{IPP}_{S}(t)+\overline{IPN}_{S}(t)}{\overline{IP}(t)}, \end{array} $$

14 $$\begin{array}{@{}rcl@{}}  MI(t)&=&\frac{\overline{IPP}_{M}(t)+\overline{IPN}_{M}(t)}{\overline{IP}(t)}. \end{array} $$

We emphasize that such indexes follow quite complex temporal patterns as soon as one deviates from the ideal situation of a population of self-renewing asynchronous cells distributed uniformly along the cell cycle. In this ideal case, the mitotic index is a constant and can be accurately estimated from the relative duration of the mitosis with respect to the whole cycle duration. Even in the still simple case of an exponentially growing cell population, the MI is subject to periodic fluctuations (with a period equaling the cell cycle duration) and the relation between the MI and the relative M phase duration becomes loglinear. Things get rapidly more complicated and intuitively unpredictable when one accounts for events leading to cell cycle exit (due for instance to quiescence, apoptosis or terminal differentiation).

We refer the interested reader to the thorough study performed in [36] on the MI dynamic pattern. We also detail in Additional file 1 the computation of these indexes in the simplified situation where both *γ* and *F*_*AP*_(*t*) are taken constant. LI, resp. MI, can then be expressed as explicit functions of the cell cycle duration, and phase S, resp. M, duration.

To sum up, the IP dynamics spans three scales:

- Cell densities *IPP*(*t*,*a*) and *IPN*(*t*,*a*) are defined on the lowest (local) level;
- Partial cell numbers $\overline {IPP}_{P}(t)$, $\overline {IPN}_{P}(t)$, $\overline {IP}_{P}(t)$ (with *P*∈{*G*1,*S*,*G*2,*M*}), and the related cell kinetics indexes are defined on the intermediate (semi-local) level;
- Whole cell numbers $\overline {IPP}(t),\overline {IPN}(t)$ and $\overline {IP}(t)$ are defined on the highest (global) level.

### Acquisition and exploitation of experimental data

**Fig. 3:**
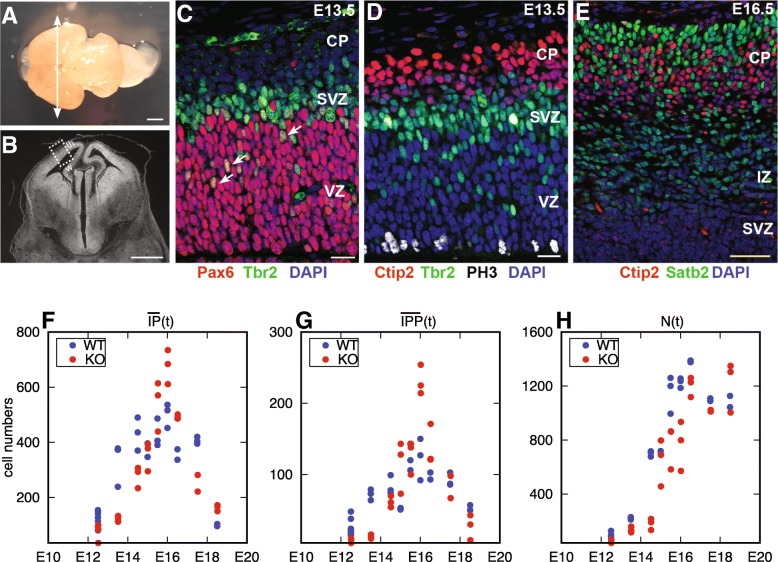
Experimental cell counts: protocol and results **a** Dorsal view of a wild-type E16.5 mouse brain. The level of the coronal sections used in this study is indicated by a white double arrow. **b** Coronal cryosection of an E13.5 mouse brain corresponding to the level of section shown in **a**. The window used for the quantification is framed. w: width; th: thickness. **c**-**e** Confocal scanning micrographs (1 *μ*m single optical section) of coronal sections of the dorsal telencephalon at E13.5 (**c**&**d**) and E16.5 (**e**). **c** Immunostaining with antibodies against Pax6 and Tbr2. DAPI counterstaining is used to label all the nuclei. White arrows show a selection of Pax 6^+^*Tbr*2^+^ nuclei (IPPs). **d** Immunostaining with antibodies against Tbr2, PH3 (to label cells in M-phase), Ctip2 (to label neurons). **e** Immunostaining with antibodies against Ctip2 (to label deep layer neurons) and Satb2 (to label upper layer neurons). IZ: Intermediate Zone. Scale bars: 1 mm in **a**, **b**, 50 *μ*m in **c**, **d**, 150 *μ*m in **e**. **f**-**h** Experimental cell counts for control (WT, blue dots) and *Ftm*^−/−^ (KO, red dots) samples

To obtain data to fuel the model, we quantified three cell populations during cortical neurogenesis: APs, IPs, and Ns. For this quantification, we performed immunofluorescence on thin sections, with a combination of markers [37–39] (Table 2, Additional file 3 and Fig. 3). The counting strategy is detailed in Methods. In order to estimate the proportion of IPPs and IPNs, we quantified the number of Pax 6^+^*Tbr*2^+^ progenitors, proposed previously to represent the pool of IPPs [16, 40]. Figure 3f-h represents the experimental cell numbers in wild-type (blue) and *Ftm*^−/−^ (red) cortices at different developmental stages. The three panels correspond respectively to the IPs (F), IPPs (G) and neurons (H). Experimental AP numbers were not used for the model calibration, since their proliferative divisions increase not only cell crowding in the pseudo-stratified VZ but also the surface of the VZ [41].

**Table 2:** Cell types and marker combinations used for their quantification

Cortical cell	Stage	Marker combination	ref
Apical progenitors (APs)	E12.5-E18.5	Pax6^+^, Tbr2^-^	[37]
Intermediate progenitors (IPs)	E12.5-E18.5	Tbr2^+^, Ctip2^-^	[37]
Proliferative IPs (IPPs)	E12.5-E18.5	Pax6^+^Tbr2^+^	[40]
Neurons	E12.5	Ctip2^+^, Tbr2^-^	[38]
	E13.5-E18.5	(Ctip2^+^, Satb2^-^) + (Ctip2^+^, Satb2^+^)	[39]

### Model simulation and parameter calibration

To simulate the model equations we have to choose specific formulations for the rate functions *β*(*t*) (rate of IPgenic AP divisions) and *γ*(*t*) (rate of IPPgenic AP divisions over total IPgenic AP divisions) and the AP inflow rate *F*_*AP*_(*t*).

The formulation of *β*(*t*) and *γ*(*t*) results from a combination of a priori biological knowledge and *a posteriori* confrontation to data. First, *β*(*t*) and *γ*(*t*) are proportions (of a given division type), so that they are naturally bounded from below and above. A constant or saturated expression is thus expected, and in both cases the parameter search range is restricted to [ 0,1]. In addition, since the sequence of proliferation and differentiation of neural progenitors in the cortex is subject to several intrinsic and extrinsic dynamical processes (review in [42] and discussed in more details in the Discussion) it seems natural to use time-dependent functions. The choice of a sigmoid shape is then pretty natural, and, in addition, it is convenient from a numerical viewpoint. Indeed, the dynamic signaling cues all act through molecular mechanisms, so that the choice can be oriented towards an enzymatic, dose-effect like curve, as those used for biochemical networks. We also assume a monotonic trend in the temporal changes of *β*(*t*) and *γ*(*t*), since they are induced by relatively low processes (as gene expression) with respect to the duration of the mid-neurogenesis time window. It is worth mentioning that the direction of this trend was not imposed a priori. Furthermore, the “effective” pattern controlling the entering flux into the IPP compartment is given by boundary condition (3) involving *F*_*AP*_(*t*) as well, so that this flux is non monotonic.

Each function involves four parameters *β*_0_ (respectively *γ*_0_), *β*_1_ (resp. *γ*_1_) corresponding to their value at respectively −*∞* and +*∞*, and *s*_*β*_ (resp. *s*_*γ*_), *t*_*β*_ (resp. *t*_*γ*_) which control respectively the slope and location of the inflexion point

15 $$ {}{\begin{aligned} \beta(t)=\beta_1+\frac{\beta_0-\beta_{1}}{1+ e^{s_{\beta}(t-t_{\beta})}}, \quad,\quad \gamma(t)=\gamma_1+\frac{\gamma_0-\gamma_{1}}{1+ e^{s_{\gamma}(t-t_{\gamma})}}, \end{aligned}}  $$

with *s*_*β*_>0 and *s*_*γ*_>0. The formulation of *F*_*AP*_(*t*) should take into account both the initial increase in the number of APs engaged in neurogenesis (hence the increase in IPgenic and possibly neurogenic divisions of APs at the expense of proliferative divisions), and the exhaustion of the AP pool through the end of neurogenesis. Thus, we need to define a function whose shape would include both an ascending and descending part, possibly separated by a plateau. Note that, in contrast to *β*(*t*) and *γ*(*t*) which are dimensionless, *F*_*AP*_(*t*) has the physical unit of number of cells per hour, i.e. density. On the biological ground, in addition to the rate of asymmetric divisions or glial transformation, this number can be further altered by the occurrence of AP cell death and changes in the AP cell cycle duration. To preserve the possibility of subsequent interpretation in terms of division type and glial transformation, we combine a constant scale factor with two rate-like rise and decay functions (hence bounded between 0 and 1):

16 $$\begin{array}{@{}rcl@{}} F_{AP}(t)=K_{AP}\frac{e^{s_+(t-t_+)}}{\left(1+e^{s_+(t-t_+)}\right)\left(1+e^{s_-(t-t_-)}\right)} \end{array} $$

where *K*_*AP*_ is the scale factor. Parameters *t*_+_ and *t*_−_ denote the time at the middle of the ascending and descending parts. Parameters *s*_+_ and *s*_−_ control the absolute value of the corresponding slopes. We thus ensure that for *t*≪*t*_+_<*t*_−_ and *t*≫*t*_−_>*t*_+_, *F*_*AP*_(*t*)→0. As a consequence, in the model the final neuron number is achieved when all other cell compartments have been emptied.

Note that with this choice for *β*(*t*),*γ*(*t*) and *F*_*AP*_(*t*), the resulting cell inflows ruled by boundary conditions (3),(4), and (5), have a similar shape as the *erf(t)* functions used in [16] to model the transitions between different cell types.

#### Control of the neuronal Pool

Before proceeding to the model calibration, we illustrate here, in the simplified framework of constant rates, the effect of *β* (impacting the indirect neurogenesis) and *γ* (impacting the IPP production) on the size of the final neuronal pool as well as the transient changes in the neuron number. For each AP entering neurogenesis, we can compute the global neuronal yield from the relative proportions of each division type:

17 $$ {}{\begin{aligned} r^{N}_{AP}=(1-\beta)+2\beta(1-\gamma) +4\beta\gamma =1+\beta(1+2\gamma). \end{aligned}}  $$

**Fig. 4:**
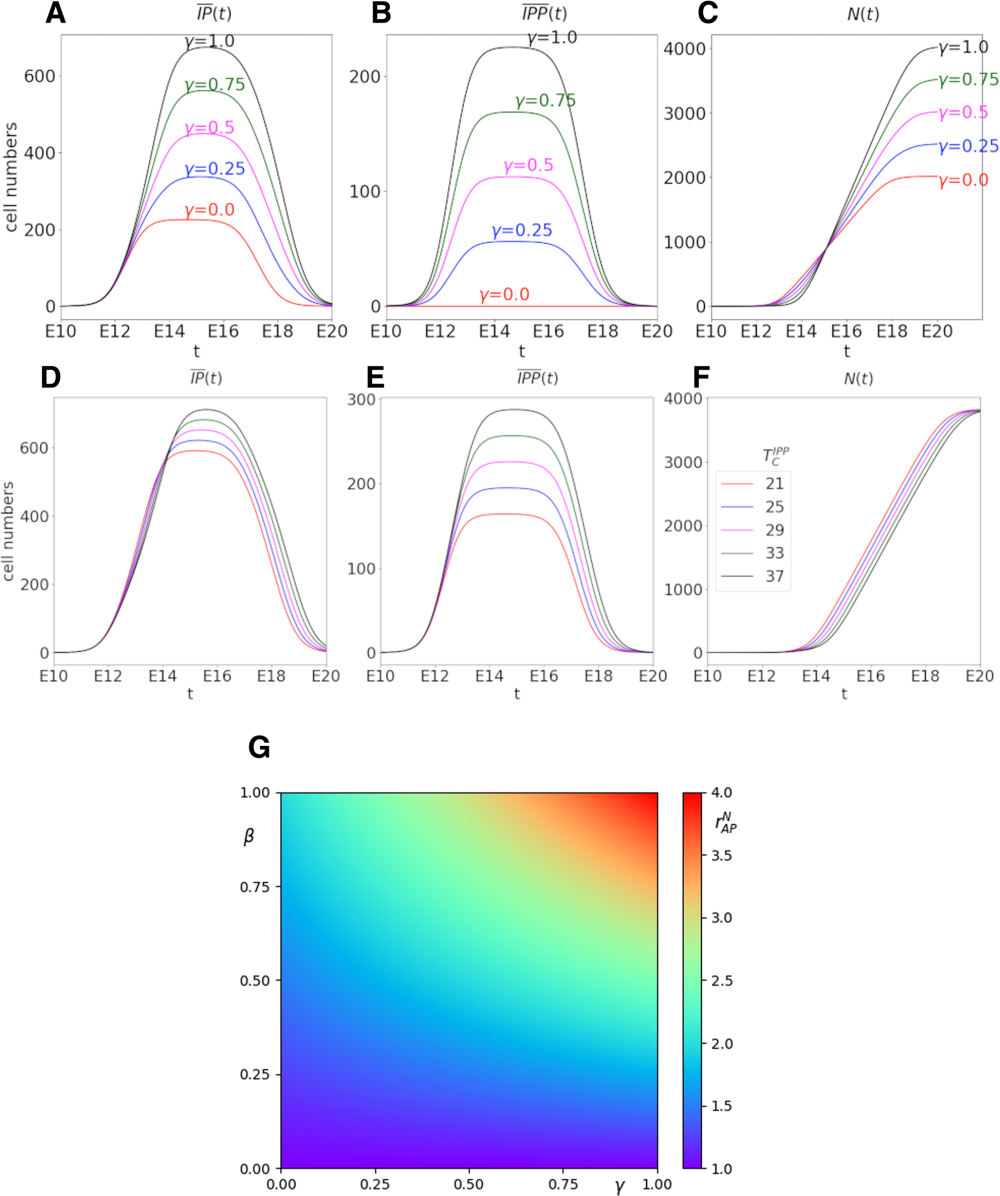
Influence of *γ* on $\overline {IP}(t)$ (panel **a**), $\overline {IPP}(t)$ (panel **b**) and *N*(*t*) (panel **c**). No direct neurogenesis and same cycle duration (*T*_*C*_=26.2) for both type of IPs, parameters for *F*_*AP*_(*t*) as in line #3 of Table 4. Influence of $T_{C}^{IPP}$ on $\overline {IP}(t)$ (panel **d**), $\overline {IPP}(t)$ (panel **e**) and *N*(*t*) (panel **f**) for constant *γ*(*t*)=0.9. **g** Color map of the ratio of total neuron production over total AP entering neurogenesis, as a function of *γ* and *β*, for constant coefficients. The color code for $r^{N}_{AP}=1+\beta (1+2\gamma)$ is indicated on the right

$r^{N}_{AP}$ would equal 1 if there was only direct neurogenesis from APs (*β*=0), 2 if there was no direct neurogenesis and no IP undergoing two division cycles (*β*=1, *γ*=0), and 4 if there was no direct neurogenesis and all IPs underwent two division cycles (*β*=1, *γ*=1). Beyond these schematic situations, $r^{N}_{AP}$ can take any value between 1 and 4, and remains unchanged on isovalues of *β*(1+2*γ*) as shown in Fig. 4g. Figure 4 displays the changes in the numbers of IPs (Fig. 4a), IPPs (Fig. 4b) and neurons (Fig. 4c), as a function of time, for different values of *γ* and in the absence of direct neurogenesis (*β*=1). As expected the maximum and cumulated numbers of IPPs and IPs increase with *γ*, and consequently the final number of neurons. Increasing *γ* also delays the onset of neuron production. In panels D, E and F, we now keep *γ* constant, as well as *β*=1, and let the IPP cell cycle duration vary from 21 to 37 h (the reference duration being 29.4 h). A high value has been chosen for *γ* (0.9) in order to get a pronounced effect of the IPP cell cycle duration on the outputs. Shortening the cycle advances the production of neurons, since IPPs exit the cell cycle and divide into IPNs earlier.

These simulations illustrate how the proportion of IPPs tunes the amplifying factor of neuron generation, as defined by (17). In contrast, the duration of the IPP cell cycle impacts the kinetics of neuron formation without affecting the final neuron number.

#### Fitting results and parameter calibration on experimental data

A priori information can be used for some of the model parameters, such as the durations of the cell cycle phases (gathered in Table 3) provided in [6], a study which provides a comprehensive description of the cell cycle in each progenitor type depending on the fate of its progeny. In order to distinguish IPPs and IPNs, the authors made use of the *T*is21-GFP transgenic mouse line, in which GFP is selectively expressed in neuron-generating progenitors [7]. Our experimental data consist in cell counts of intermediate progenitors and neurons, on a daily basis between E12 and E18. Double stained Pax 6^+^*Tbr*2^+^ cells are considered as a rough estimate of IPPs [16, 43].

**Table 3:** Durations of the IPP and IPN cell cycle phases at E14.5 taken from Arai et al. [6]

Durations of cell cycle phases					
	*T*_*C*_	*T*_*S*_	*T*_*G*2_	*T*_*M*_	*T*_*G*1_
IPPs	29.4	6.4	1.6	0.5	20.9
IPNs	26.2	2.8	1.6	0.5	21.3

Time are given in hours (h)

We thus have three series of datasets corresponding to the model outputs *IPP*(*t*), *IP*(*t*) and *N*(*t*), whose amplitude ranges are quite contrasted. Indeed, the maximal number of neurons is clearly greater than that of intermediate progenitors, since neurons accumulate over time, while the dynamics of intermediate progenitors is transient. In the same way, the maximal number of *IPP* is smaller than that of *IP*. To fit the model parameters, it is thus natural to design a multi-objective function *J*(*p*) balancing rigorously the contribution of the different datasets, as detailed in the “Methods” section.

We minimize *J*(*p*) over the parameter set

18 $$\begin{array}{@{}rcl@{}} {\cal P}&=&\left\{ p=(K_{AP}, s_+,t_+, s_-,t_-, \gamma_0, \gamma_1, s_{\gamma}, t_{\gamma}), \right.\\  &&\left. p_{i}\in [p_{\min}^{i},p_{\max}^{i}], i=1,\ldots,9\right\}. \end{array} $$

The size of this set is reduced to nine parameters, as we have set *β* to 1, which amounts to neglecting direct neurogenesis. This choice was motivated by preliminary optimization trials, in which the estimated value of *β*(*t*) systematically happened to be non time-varying and close to 1.

We performed several simulations with different sets of parameters obtained from the minimization of *J*(*p*). The values of the criterion and parameter sets are gathered in Table 4. For each simulation, we display, in addition to the model outputs,

- the coefficients *C*_*IP*_, *C*_*N*_ and *C*_*IPP*_ that indicate which of the three datasets entered the calibration. They are all equal to 1/3 if all three datasets are taken into account in the calibration. If *C*_*IPP*_=0, only the *IP* and *N* datasets enter the calibration with an equal weight *C*_*IP*_=*C*_*N*_=1/2.
- the elementary fit values *J*_*IP*_(*p*), *J*_*IPP*_(*p*) and *J*_*N*_(*p*) on either of the three datasets *IP*, *IPP* and *N*, which are combined to compute the global criterion *J*(*p*).
- the ratio between the number of produced neurons and the number of APs which have entered neurogenesis in the course of the simulation, from initial time *t*_0_=0 to stopping time *t*=*T*, which we set equal to E20 in the numerical simulations
  19 $$\begin{array}{@{}rcl@{}} r_{AP}^{N}(T)=\frac{N(T)}{\int_{0}^{T}F_{AP}(t) dt}{,} \end{array} $$
  which can also be directly estimated from the extension of formula (17) with time-dependent rates:
  $$\begin{array}{@{}rcl@{}} r_{AP}^{N}(T)=\frac{1}{T}\int_{0}^{T}{1+ \beta(t) +2\beta(t)\gamma(t)dt}{.} \end{array} $$

**Table 4:** The four simulations, illustrated in Figs. 5 and 6, are performed with parameters fitted on wild type (WT) or *Ftm* mutant (KO) data, taking a cell cycle duration of 29.4h for the IPP cell type as in [6]

	

The *γ*(*t*) rate is either a constant (cte) or a sigmoid function of time (var), and the datasets used in the fit (27) include IPs and Ns in all cases, and additional data on IPPs in cases 3 and 4. The parameter values obtained from optimization are highlighted in blue. The corresponding a priori criterion values are highlighted in red. The values of the criterion recomputed a posteriori with adding (upper line) or removing (lower line) the IPP dataset are left in black

**Fig. 5:**
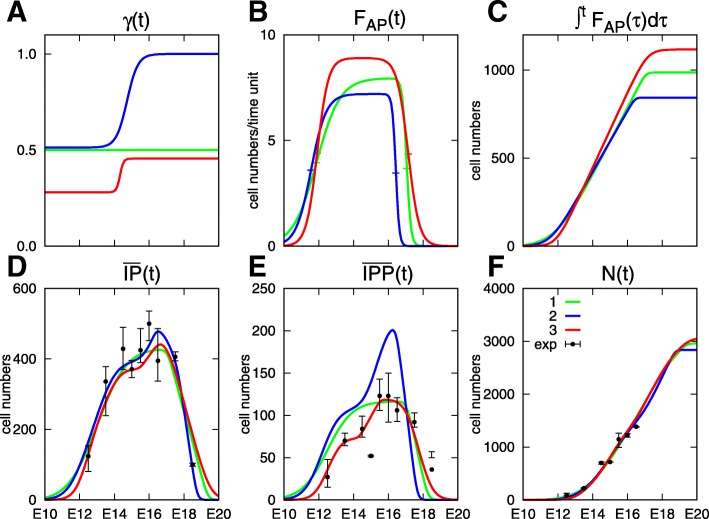
Optimization results on the control dataset. Coefficients and outputs of the model for the three parameter sets in Table 4, superimposed on experimental values represented by their mean and global range (in black). Green (*#*1) and blue (*#*2) lines correspond to a fit with neurons and IPs, red lines (*#*3) correspond to the fit including IPPs. Green lines correspond to the fit with *γ*(*t*) kept constant. Panel **a** displays the division rate *γ*(*t*). Panel **b** displays the input flux of AP cells *F*_*AP*_(*t*), the + signs show the location of the inflexion times *t*_−_ and *t*_+_. Panel **c** displays the cumulative number of AP cells $\int _{0}^{t}F_{AP}(\tau)d\tau $. Panel **d** displays $\overline {IP}(t)$ computed by the model (7). Panel **e** displays $\overline {IPP}(t)$ computed by the model (6). Panel **f** displays *N*(*t*) computed by the model (8). The value of the ratio $r^{N}_{AP}$ for the three simulations is 3. (scenario 1 in green), 3.37 (scenario 2 in blue) and 2.73 (scenario 3 in red)

We first adjusted the model parameters from the neuron and total IP numbers of the WT dataset, with *C*_*IPP*_=0 and either a constant (scenario 1, green line in Fig. 5) or time-varying *γ*(*t*) (scenario 2, blue line). We then included the IPP cell number in the dataset keeping a time-varying *γ*(*t*) (*C*_*IPP*_=1/3, scenario 3, red line).

All three scenarios lead to rather similar patterns for the neuron curve. In scenario 2, the absence of direct production of IPNs from APs during the final part of neurogenesis (*γ*=1 after E16), which could be expected to lead to delayed N production, is actually compensated for by a premature decay of the AP input flux, compared to scenario 1 and 3 (panel B).

In contrast, these scenarios clearly differ as far as fitting IP numbers. Scenario 1 and 2 both fail to capture correctly the initial rise in IPs, which occurs too early with respect to the experimental data. Thanks to the introduction of the time variability in *γ*, scenario 2 does capture the decay in IPs, which improves the fit on IPs and neurons (it is reduced to the fourth of the value obtained with scenario 1). Scenario 3 is rather close to scenario 1 for Ns (even if the neuronal yield $r^{N}_{AP}$ is slightly lowered) and total IPs. Yet, as expected, the fitting to IPP data is drastically improved and involves a downward translation of the *γ*(*t*) curve. There remains a shift to the right along the descending part of the IPP curve, which can be explained by the discrepancy observed between the experimental IP and IPP datasets (WT IPP values exceed IP values at E18.5, see panels F and G of Fig. 3) at the end of neurogenesis. As discussed elsewhere, IPP are identified as Pax 6^+^*Tbr*2^+^ cells; this marker appears to be less reliable during late neurogenesis.

The simulations displayed in Fig. 5 can be reproduced with an Ipython notebook, CEMONE (CEll based MOdel of NEurogenesis) which can be either downloaded from https://github.com/letsop/cemone or directly run on https://mybinder.org/v2/gh/letsop/cemone/master.

**Fig. 6:**
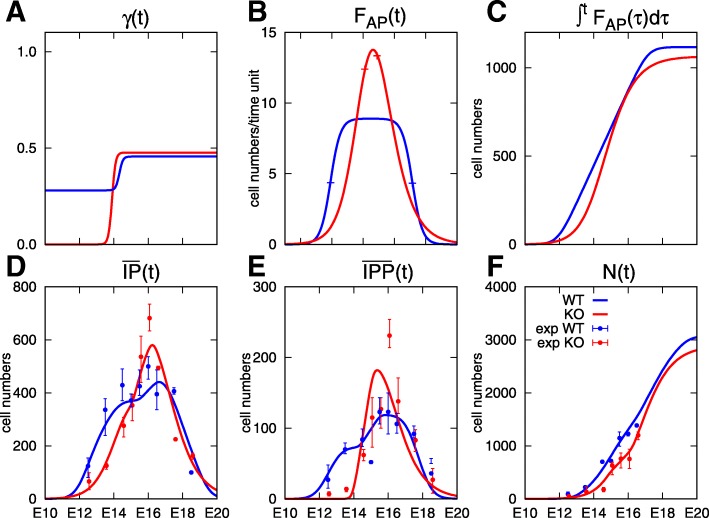
Comparison of wild-type (WT, in blue) and *Ftm*^−/−^ (KO, in red) results. Coefficients and outputs of the model for the parameter sets #3 and #4 in Table 4, superimposed on experimental values represented by their mean and global range. Panel **a** displays IPPgenic division rate of APs, *γ*(*t*). Panel **b** displays the input flux of AP cells, *F*_*AP*_(*t*), the + signs show the location of the inflexion times *t*_−_ and *t*_+_. Panel **c** displays the cumulative number of AP cells entering neurogenesis, $\int _{0}^{t}F_{AP}(\tau)d\tau $. Panel **d** displays $\overline {IP}(t)$ computed by the model (7). Panel **e** displays the IPP number curve $\overline {IPP}(t)$ computed by the model (6). Panel **f** displays the neuron number curve *N*(*t*) computed by the model (8). The blue (respectively red) dots and vertical bars correspond to the mean values and whole range of the control (resp. mutant) experimental dataset. The ratio $r^{N}_{AP}=2.73$ for the control and $r^{N}_{AP}=2.65$ for the mutant

From the results obtained on the control dataset, we directly performed the parameter optimization on the mutant dataset in a situation similar to scenario 3 (time-dependent *γ*(*t*) and *C*_*IPP*_=1/3, scenario 4 in Table 4). We compare the control and mutant results in Fig. 6. The differences observed in the experimental datasets mainly concern the IP (and IPP) peak, which is higher and narrower, and also occurs later in mutant mice compared to control. This difference can be explained by the observed alteration in the patterns of *γ*(*t*) and *F*_*AP*_(*t*). In the mutant case, *γ*(*t*) is not only shifted downwards, to the point that the minimal value sticks to the zero floor at the beginning of neurogenesis, but also its shape is distorted, with a much sharper increase from 0 to 0.48 just before E14 (panel A). In the meantime *γ*(*t*) rises from 0.28 to 0.46 in the control case. *F*_*AP*_(*t*) also changes in a very different manner (panel B). Rather than reaching a plateau, hence being almost constant during most of the neurogenesis period (from E14 to E16), the flux of committed APs in the mutant case is both peak-shaped and condensed in time. In the control case, the increase in the cumulated flux is close to linear and reaches a steady value around E17. In the mutant case, the curve is first sublinear and then becomes superlinear, around E16, where it matches the corresponding value for the control. Nevertheless, the total number of APs entering neurogenesis (panel F) is very similar, and the neuronal yield is almost as high in mutant (2.65) as in control (2.73). Note that the changes in the cumulated AP flux and neurons look similar in controls and mutants, yet they are not identical. In the model, the IP dynamics operate as a transfer function from the AP input to the N output. As observed in the case of constant rates, the proportion of IPPs tunes the amplification factor of neuron production, while the durations of the IPP and IPN cell cycles impact the kinetics of neuron apparition and further increase.

In both the control and mutant cases, we had to optimize a fitting criterion with a relatively large number of parameters, entering the definition of *F*_*AP*_(*t*) and *γ*(*t*). Notwithstanding the relatively high dimension of the parameter space, the multi-criterion based optimization procedure following the lines of [44] has allowed us to take advantage of our multiple experimental datasets. In the case of the *Ftm* mutant, our simulations suggest that the perturbed dynamics of neurogenesis cannot be accounted for by a clear difference in IP proliferation mode or division rate. The model allows us to predict that obtaining a similar neuronal output with a shorter neurogenesis period requires an important and transient inflow of APs (*F*_*AP*_).

Given the shape of *γ*(*t*) on panel a of Fig. 6, one could argue that a piece-wise constant function could have been chosen for *γ*(*t*). Yet, this switch-like pattern was not particularly expected a priori, and we did not consider that it was worth reformulating this function at this stage, given that the transition was nevertheless smoother in the control situation compared to the really sharp transition in the mutant. Also, the statistical estimation of point switching times is a rather tricky issue especially in a context of low time-resolution data such as ours.

### Derivation of cell kinetics indexes and associated quantitative information on proliferative versus neurogenic cell populations

In our experimental setup, we have considered, following [16] and [43], that the specific combination of Pax6 and Tbr2 staining can help discriminate IPPs from IPNs. Other experimental markers derived from cell kinetics principles have been proposed in the literature [45]. The mitotic index (MI; ratio of cells in M phase) and the labeling index (LI; ratio of cells in S phase) in a given progenitor population can be assessed with experimental labeling. This can be achieved for instance using immunoreactivity for phosphorylated Histone H3 (to label M phase), and incorporation into the DNA of nucleoside analogs such as BrdU, EdU or IddU followed by their immunofluorescence detection (to label S phase). We now explain how to compute such indexes in silico and to use the model outputs to assess their robustness to either extrinsic (such as the time chosen to perform the measurements) or intrinsic (such as the duration of cell cycle and cell cycle phases) factors.

**Ratios based on the mitotic index** Several studies have intended to quantify neurogenic mitoses in AP and IP populations, using different approaches [6, 16, 46]. Here we show that we can use the model to assess the ratio of IPs and APs committed to a neurogenic division.

The ratio *ψ*(*t*) of neurogenic IP mitoses can be computed exactly from the direct counting of mitotic cells (cf Eq. (12)) as

20 $$\begin{array}{@{}rcl@{}} \psi(t)=\frac{\overline{IPN}_{M}(t)}{\overline{IPP}_{M}(t)+\overline{IPN}_{M}(t)}. \end{array} $$

Since phase M is relatively short compared to the cell cycle duration, we can also estimate *ψ*(*t*) from the fluxes of cells getting out of either the IPP or IPN cell cycles

21 $$\begin{array}{@{}rcl@{}} \psi(t)&\approx &\frac{IPN(t,T^{IPN})}{IPN\left(t,T^{IPN}\right)+IPP\left(t,T^{IPP}\right)}. \end{array} $$

These quantities can be computed using the method of characteristics

$$\begin{array}{@{}rcl@{}} \psi(t)&\approx &\frac{IPN(t-T^{IPN},0)}{IPN\left(t-T^{IPN},0\right)+IPP\left(t-T^{IPP},0\right)}. \end{array} $$

The incoming cell flux into the IPN cell cycle is composed of the contribution of both APs and IPgenic IPPs

22 $$ {}{\begin{aligned} IPN(t,0)&=F_{AP}(t)(1-\gamma(t))\beta(t)+2IPP\left(t,T^{IPP}\right)\\ &=F_{AP}(t)(1-\gamma(t))\beta(t)+2IPP\left(t-T^{IPP},0\right) \end{aligned}}  $$

while only AP divisions contribute to the incoming cell flux into the IPP cell cycle

23 $$\begin{array}{@{}rcl@{}} IPP(t,0)=F_{AP}(t)\gamma(t)\beta(t). \end{array} $$

Even if we do not represent as such the dynamics of APs, the model also allows us to compute the number of neurogenic AP mitoses from the cell fluxes getting out of the AP compartment (following the same approximation based on the short duration of phase M as above).

As shown in panels c and f of Additional file 1: Figure S1, the agreement between the approximation obtained by plugging (22) and (23) into (21) and the exact computation (20) is very good as soon as the number of mitotic cells is large enough, which guarantees that the denominator of (20) is not too close to zero (which can happen when the cycles get emptied from their cells).

Given a set of parameters estimated from data dealing only with IPs (possibly including IPPs) and neurons, we can derive (among several other model outputs), the neurogenic fraction. The computation of the neurogenic fraction with the parameters selected from our optimization procedure can thus be considered as a prediction of the model. Interestingly, this prediction is consistent with the results obtained in [46]. In this work, the authors have taken advantage of the *T*is21-GFP staining in combination with Pax6 or Tbr2, in addition to PH3, to identify the neurogenic fate of APs and IPs in a specific way. They have shown that a significant proportion of IP mitoses are not neurogenic and that very few AP mitoses are neurogenic, which corroborates the very low, or even neglectible value of direct neurogenesis from AP in our setup.

**Ratios based on the labeling index** *Experimental rationale.* Cumulative labeling [6] or double labeling [47] by incorporation of nucleoside analogs is classically used to assess the duration of S phase. Another application of S phase double labeling was proposed to try to discriminate, within a given progenitor cell population, cells undergoing a second round of division from cells progressing along their first cell cycle within this cell type [45]. In this case, the delay between the administration of both dyes is much longer, on the order of the duration of a cell cycle (and should be finely tuned as explained below), and cells are labeled sequentially from one generation to the next (only daughter cells can be double-labeled). In more details, the experimental protocol consists in administering first BrdU at a given time *t*_1_, and then, after an appropriate (and long) delay *t*_2_−*t*_1_, EdU. After *t*_2_, the number of double-labeled (*BrdU*^+^*EdU*^+^) cells is an estimate of the number of cells that have gone through two rounds of S phases since *t*_1_, while single-labeled (*Brdu*^−^*Edu*^+^) cells were not in S phase at the time of BrdU injection.

In silico *assessment.* Here we focus on the in silico assessment of double-labeled cells in the case of the IP population, and highlight its sensitivity to extrinsic (choice of the delay between BrdU and EdU administration) and intrinsic (differences in S phase duration between IPP and IPN) factors.

As in the case of the ratios derived from mitotic indexes, one great advantage of the modeling approach is to have access to the reference (“true”, computed) value, and to be able to compare the estimated values with respect to this reference.

The number of IPPs in S phase, hence labeled with BrdU, at *t*_1_ is given by:

24 $$\begin{array}{@{}rcl@{}}\overline{IPP}_{S}(t_1)=\int_{T^{IPP}_{G1}}^{T^{IPP}_{G1}+T^{IPP}_{S}} IPP(t_{1},a)da \,.\end{array} $$

Hence, the reference value is 2 $\overline {IPP}_{S}(t_{1})$ (factor 2 accounts for the occurrence of mitosis in *BrdU*^+^ cells during the *t*_2_−*t*_1_ interval). Note again that this number is not available as such in the experimental protocol, and is rather approximated from the number of *BrdU*^+^*EdU*^+^ cells.

On the other hand, at *t*_2_, the total number of IPs labeled with EdU corresponds to the number of IPs progressing along S phase. Amongst these, only IPNs may have undergone a former cell cycle as IPPs and are susceptible to be double labeled. We derive in the Methods section, the cell number $\overline {IPN}^{\star }_{S}(t_{2})$ (Eq. (34)) mimicking the experimental values of double *Brdu*^+^*EdU*^+^ labeled cells as a function of the *t*_2_−*t*_1_ delay and durations of cell cycle phases.

The computation of $\overline {IPN}^{\star }_{S}(t_{2})$ amounts to removing all possibly “false positive” double labeled cells, which is a modeling issue (since we cannot stain cells in the model). In the next session, we will also deal with “false negative” cells, which is really an experimental issue, that may lead to underestimate the number of IPPs in S phase at *t*_1_.

*Optimal protocol design : sensitivity to the delay and S phase durations.* Now, we can compare the experimental-like estimated value, $\overline {IPN}_{S}^{\star }(t_{2})$, with the reference value, 2 $\overline {IPP}_{S}(t_{1})$, according to the chosen *t*_2_−*t*_1_ delay and the difference in S phase durations between IPPs and IPNs. In consistency with biological knowledge, we restrict our study to the case when $T^{IPP}_{S}\geq T^{IPN}_{S}$, yet it can be easily generalized to the opposite case. The quality of the estimation can be summarized by the proportion of detected cells (positive predictive value) *ρ*:

25 $$ \rho={\frac{\overline{IPN}_{S}^{\star}(t_{2})}{2\overline{IPP}_{S}(t_{1})}}.  $$

1. If $t_{2}-t_{1}\leq T^{IPP}_{G2}+T^{IPP}_{M}+T^{IPN}_{G1}$, then no BrdU+ cells has had the time to reach S phase by *t*_2_, and *ρ*=0%;
2. If $T^{IPP}_{G2}+T^{IPP}_{M}+T^{IPN}_{G1}< t_{2}-t_{1}<T^{IPP}_{G2}+T^{IPP}_{M}+T^{IPN}_{G1}+T^{IPN}_{S}$ then a subpart of Brdu+ cells lie within a new S phase and will be detected as BrdU+EdU+ cells. The value of *ρ* increases as linearly *t*_2_−*t*_1_ increases;
3. If $T^{IPP}_{G2}+T^{IPP}_{M}+T^{IPN}_{G1}+T^{IPN}_{S}\!< t_{2}-t_{1}<\!T^{IPP}_{G2}+T^{IPP}_{M}+T^{IPN}_{G1}+T^{IPP}_{S}$, the value of *ρ* has reached a plateau, whose height increases as the ratio $T^{IPN}_{S}/T^{IPP}_{S}$ increases, up to the maximal value of *ρ*=100% when $T^{IPP}_{S}\,=\,T^{IPN}_{S}$;
4. If $T^{IPP}_{G2}+T^{IPP}_{M}+T^{IPN}_{G1}+T^{IPP}_{S}< t_{2}-t_{1}<T^{IPP}_{G2}+T^{IPP}_{M}+T^{IPN}_{G1}+T^{IPP}_{S}+T^{IPN}_{S}$ then the value of *ρ* decreases linearly as *t*_2_−*t*_1_ increases;
5. If $t_{2}-t_{1}\geq T^{IPP}_{G2}+T^{IPP}_{M}+T^{IPN}_{G1}+T^{IPP}_{S}+T^{IPN}_{S}$, then all BrdU+ cells have overcome S phase by *t*_2_, and *ρ*=0%.

**Fig. 7:**
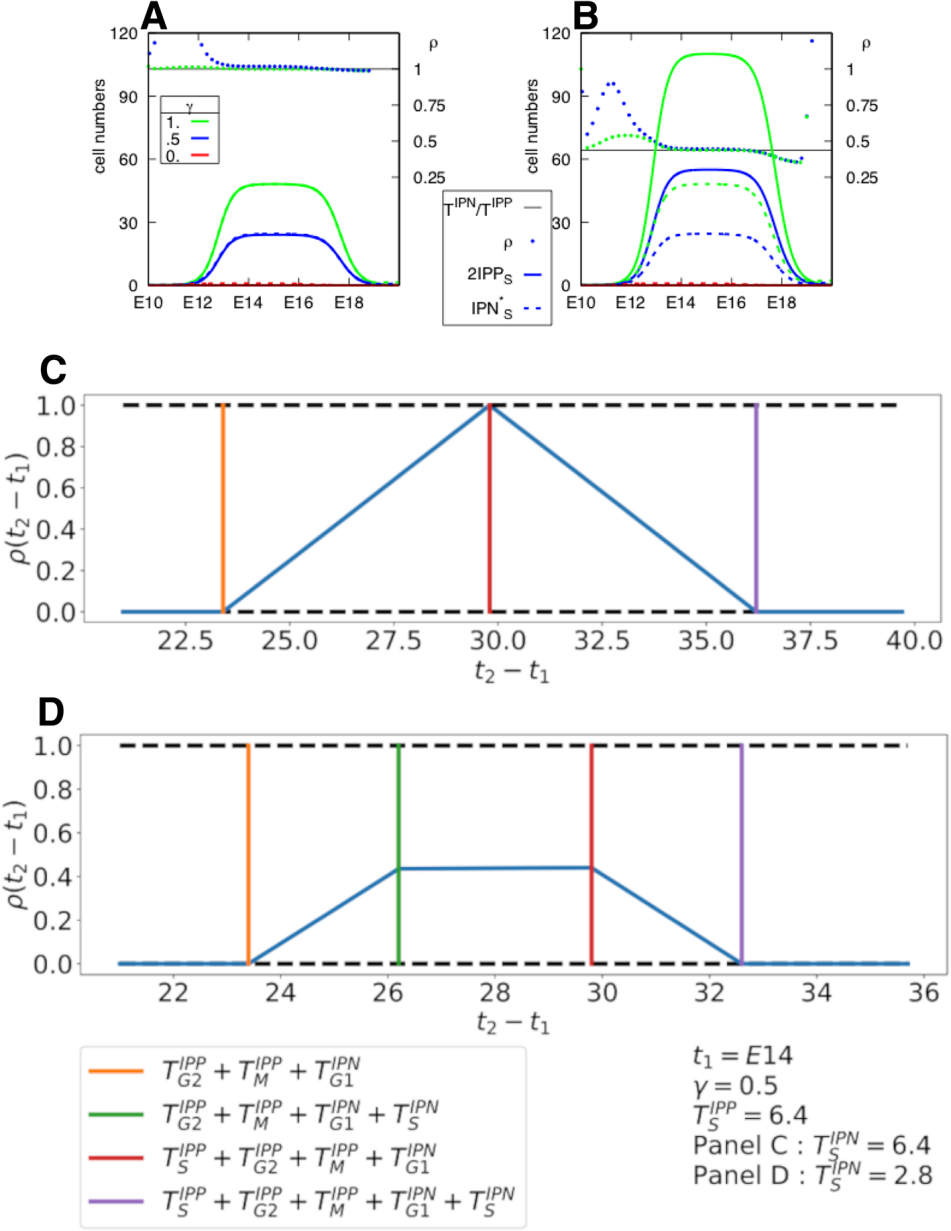
Panels **a** and **b**: Comparison of the numbers of *IPP*s in S phase at *t*_1_ ($2\overline {IPP}_{S}(t_{1})$ (24), solid line) with their estimation from double BrdU+EdU+ labeled cells at *t*_2_ ($\overline {IPN}_{S}^{\star }(t_{2})$ (34), dash lines). We use the same durations as those of Table 3 for the phases of the IPN cell cycle and all phases except S phase of the IPP cell cycle. In panel **a**, $T^{IPP}_{S}=T^{IPN}_{S}=2.8$h, while, in panel **b**, $T^{IPP}_{S}=6.4$h and $T^{IPN}_{S}=2.8$h. The input flux *F*_*AP*_(*t*) is the same as Case (3) of Table 4. *γ*(*t*)=*cte* (0, 0.5 or 1). The ratio *ρ*(*t*) between the estimated and the true value (Eq. (25) is also plotted on each panel, according to the scale ticked on the right axis, alongside the theoretical value $T^{IPN}_{S}/T^{IPP}_{S}$ (in black). Panels **c** and **d**: Sensitivity of the protocol to the delay and S phase duration. The two panels display the ratio *ρ*(*t*_2_−*t*_1_) (Eq. (25)) as a function of *t*_2_−*t*_1_ for *t*_1_=*E*14 and *γ*(*t*)=0.5=*cte*

In Fig. 7a and b, we illustrate the comparison between the true value (2 $\overline {IPP}_{S}(t_{1})$) and the estimated one $\left (\overline {IPN}_{S}^{\star }(t_{2})\right)$, in the case of an optimal choice of the delay *t*_2_−*t*_1_ (case 3 above). In panel A, the duration of S phase is the same for IPPs and IPNs, and, as expected, *ρ*=1 (notwithstanding some discards due to numerical approximations). In panel B, the duration of phase S of IPP exceeds that of IPN, and consequently *ρ* is lower than 1. In Fig. 7c and d we show the changes occurring in *ρ* according to the delay *t*_2_−*t*_1_ in the whole range covering cases 1 to case 4 above.

The computations have been done in the case of a pulse-mode labeling (instantaneous action). Actually, there is some remanence in the labeling, which could be accounted for by computing the index on a cumulated time interval adjusted to the dye duration effect. Yet, in practice, this amounts to lengthening the apparent duration of the corresponding S phases, and the reasoning remains exactly the same. A practical fallout of this remark is that, in the case when $T^{IPP}_{S}\geq T^{IPN}_{S}$, we can recommend to extend the administration of EdU with respect to that of BrdU.

In conclusion, our model allows us to compute cell kinetics indexes, associated with given IP and neuron temporal patterns such as shown in Figs. 5 and 6. The dynamics of these indexes are complicated and non intuitive. In particular, the ordering of indexes in control and mutant reverses in time, so that we recommend to perform several time point measurements. If accurate and time detailed measurements of mitotic and labeling indexes were available, information on the cell cycle phase durations could be obtained by comparison with the model outputs (12-14), illustrated in more details in Additional file 6. We have also illustrated the potential of the model to mimic and/or plan elaborate cell kinetics experiments based on the MI and LI indexes.

## Methods

### Mice, embryo collection and staging

The Ftm mutant mouse line was produced and published by U. Rüther’s laboratory [29] and obtained by S. Schneider-Maunoury’s lab with authorization of Prof. U. Rüther, where it was maintained since 2004 and has been used for several published studies [30, 31, 48, 49]. Wild type animals for outcrossing were obtained from Janvier labs. All experimental procedures involving mice were performed in accordance with ethical guidelines of the European directive 2010/63/UE and its French application decree 2013-118, and were approved by the “Charles Darwin” local ethical committee with Project number 1382. Animals were produced and maintained in the Institut de Biologie Paris-Seine (IBPS) animal facility. The facility follows the European directive 2010/63/UE and its French application decree 2013-118 for maintenance of laboratory animals. Animal care taking was performed by a dedicated technician, supervised by the head of the facility and by the university veterinary. The facility is authorized (authorization number B 75-05-24) and regularly inspected by the Paris police Headquarters, DDPP (Direction Départementale de la Protection des Populations) service.

Adult mice were euthanized by cervical dislocation or CO2 exposure. Fetuses before E16.5 were euthanized by decapitation. Fetuses after E16.5 were anesthetized by hypothermia before transcardial perfusion (which led to death of the fetus). The *Ftm* mutant mouse line [29, 31] was maintained at the heterozygous state in the C57Bl/6j background. Heterozygous mice were incrossed in order to obtain embryos and fetuses of the desired genotypes: *Ftm*^+/+^(wild-type, wt), *Ftm*^+/−^ (heterozygotes) and *Ftm*^−/−^ (homozygote mutant). *Ftm*^+/+^ and *Ftm*^+/−^ animals were viable and fertile and their phenotype was normal [29, 31], hence they were collectively designated as controls (ctrl or WT in figures and tables). Embryonic day (E) E0.5 was defined as noon on the day of vaginal plug detection. Stage variability between litters of the same theoretical chronological age may arise as a function of the time of mating and of the potential delay in fertilization. Dissections were always performed between 3 pm and 4 pm to minimize developmental variability across litters. In order to reduce further this variability, we obtained morphometric measurements on brain sections and used anatomical criteria (Additional file 2). The histological criteria were i) the dorso-ventral extent of the 3rd ventricle (which, from E14.5 onward, progressively reduces by fusion of the left and right thalami, leaving only dorsal and ventral openings), ii) the presence of the choroid plexus in the dorsal part of the 3rd ventricle (from E14.5), iii) the thickness of the ventricular-subventricular zone in the diecenphalon (thick at E15.5, reduces afterward), iv) the folding of the hippocampus (starts at E15.5 and increases progressively) and v) the size of the caudal eminence (maximum at E16.5 and then decreases). Three adjacent brain sections were scanned using a Leica (DFC345FX) binocular. The stages of the litters were adjusted relative to each other, with a minimal 12 hour-delay between two consecutive time points.

### Immunofluorescence

Brains of embryos younger than E16.5 were dissected in cold phosphate-buffered saline (PBS) and fixed overnight at 4^∘^C in a solution of 4% paraformaldehyde in PBS (4% PFA). Fetuses at E17.5 and E18.5 were subjected to intracardial perfusion with 4% PFA; then their brains were dissected and kept in 4% PFA overnight at 4^∘^C under mild agitation. The brains were then rinsed and mounted in Optimum Cutting Temperature (OCT) and cut on a cryostat into 14 *μ*m-thick serial sections. Prior to immunofluorescence, slides were boiled with 0.1M sodium citrate buffer in a microwave oven for antigen retrieval, washed in PBS and blocked for 1h in PBS containing 0.3% Triton X-100 and 10% goat serum. Sections were immunostained by overnight incubation with primary antibodies at 4^∘^C. The following primary antibodies were used: Pax6 (Covance/eurogentec PRB-278P; 1:500), Tbr2 (Abcam 23345; 1:300) Tbr2-alexa488 (Ebioscience 53-4875; 1:400), Ctip2 (Abcam 18465; 1:300), Satb2 (Abcam 34735; 1:300). Sections were then incubated with fluorochrome-conjugated secondary antibodies (Life Technologies) for 2h at room temperature. All samples were counterstained with 1 *μ*g/mL DAPI in PBS, and mounted in Vectashield (Vector lab).

### Image acquisition and quantification

Optical sections (1 *μ*m thick) of dorsal telencephalon (Fig. 1b) were captured at 1024 density using 20x objective and 2x zoom function on a Leica confocal microscope (TCS SP5 AOBS). Cell nuclei were counted manually on a window of 387.5 *μ*m of width whose height varies according to the thickness of the cortex. For illustration, confocal images were cropped and adjusted for contrast by using Fiji and then arranged by FigureJ (plugin in Fiji) and InDesign adobe CS6.

### Cell counting strategy

Cell populations were counted in a window of constant width (“w” in Fig. 3b), which includes the whole thickness (“th” in Fig. 3b) of the cortex, at all stages (Fig. 3a-e). With this strategy, neuron numbers are underestimated as neurogenesis proceeds, since neurons, in addition to migrating radially into the cortical plate, display tangential dispersion leading to cortex growth and deformation [50–52] (panel g in Additional file 2). This is particularly true at late stages of cortical neurogenesis (from E17.5 onward), when a decrease in the number of neurons in the quantification window is observed, already mentioned in [53] and most probably due to neurite outgrowth. For this reason, we did not take into account neuron numbers at E17.5 and E18.5.

### Numerical method

The numerical method used to compute the solution of Eqs. (1-4) is based on the method of characteristics. We can solve the PDEs (1,2) analytically through the characteristic curves:

26 $$\begin{array}{@{}rcl@{}}{}\left\{\begin{array}{lcl} IPP(t,0)&=&\gamma(t)\beta(t)F_{AP}(t)\\ IPP(t,a)&=&\left\{\begin{array}{l} IPP(0,a-t)\text{ for}\ t< a<T_{C}^{IPP}\\ IPP(t-a,0)\text{ for}\ 0< a< t\end{array}\right.\\ IPN(t,0)&=&(1-\gamma(t))\beta(t)F_{AP}(t) +2 IPP\left(t,T_{C}^{IPP}\right)\\ IPN(t,a)&=&\left\{\begin{array}{l} IPN(0,a-t)\text{ for}\ t< a<T_{C}^{IPN}\\ IPN(t-a,0)\text{ for}\ 0< a< t\end{array}\right. \end{array}\right. \end{array} $$

The numerical method involves the discretization of (26) in age (space) and time. The space step *Δ*_*a*_ is chosen so that there is an integer number of meshes, *N*_*a*_, within the IPP cell cycle. Since the duration of the IPN cell cycle is smaller than that of the IPN cycle ($T_{C}^{IPP}>T_{C}^{IPN}$), not only the number of meshes $\tilde N_{a}$ is different ($\tilde N_{a}<N_{a}$), but also the latest mesh may be reduced in size:

$$\begin{array}{@{}rcl@{}} T_{C}^{IPP}&=& N_{a}\Delta_{a},\quad \text{ with }N_{a}\in\mathbb{N},\\ T_{C}^{IPN}&=&(\tilde N_a-(1-\tilde \delta))\Delta_{a}, \quad\text{with} \, \, \tilde N_{a}\in\mathbb{N}\text{ and }0\!\leq\tilde \delta<1. \end{array} $$

The time step is chosen to be equal to the space step:

$$\begin{array}{@{}rcl@{}} \Delta:=\Delta_t= \Delta_{a}. \end{array} $$

The pseudo-code in Algorithm 1 describes how to compute the discretized cell densities ${IPP}^{n}_{j}$ (respectively ${IPN}^{n}_{j}$) of IPP (respectively IPN) at times *nΔ* and ages *jΔ*

$$IPP^{n}_{j}\approx IPP(n\Delta,j\delta),\quad IPN^{n}_{j}\approx IPN(n\Delta,j\delta),$$

and how to integrate the densities over the whole domain to obtain the cell numbers, which are approximated by an order 2 quadrature. 
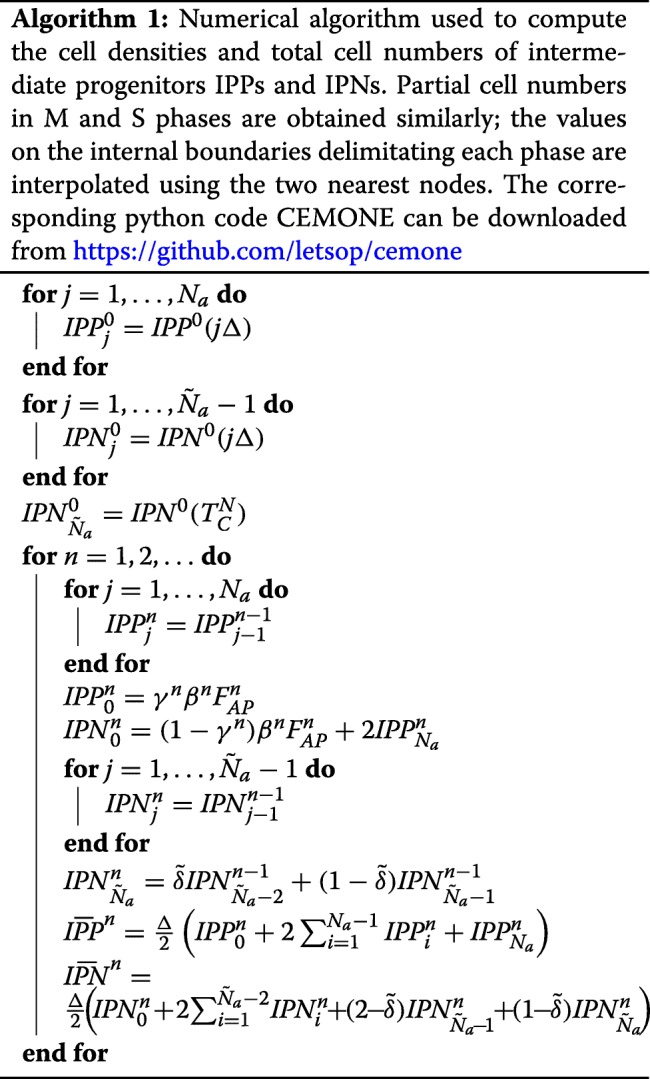


### Minimization method

In Additional file 7, we study the identifiability of the model (in the sense introduced in [54]). We show that the inverse problem associated with our model is well-posed, i.e. that one can recover *F*_*AB*_(*t*), *γ*(*t*) and *β*(*t*) from the knowledge on the time series corresponding to $\overline {IPP}(t)$, $\overline {IPN}(t)$ and *N*(*t*). The practical counterpart of the theoretical problem of identifiability consists in obtaining numerical values for the parameters of the model inputs *γ*(*t*) and *F*_*AP*_(*t*) by optimizing the fit of the model outputs with the available experimental data, which are sampled at discrete times *t*_*i*_ and subject to experimental noise and biological variability. We describe here the design of the criterion used to measure the fit, which takes into account the contrast in the amplitude ranges of the model outputs *IPP*(*t*), *IP*(*t*) and *N*(*t*). We follow the approach proposed in [44] to define a multi-objective optimization criterion *J*(*p*)

27 $$\begin{array}{@{}rcl@{}} J(p)&=& C_{IP}\frac{\left(J_{{IP}}(p)-J_{IP}^{\star}\right)^{2}}{\left(J_{{IP}}^{{\max}}-J_{IP}^{\star}\right)^{2}}+C_{IPP}\frac{\left(J_{{IPP}}(p)-J_{IPP}^{\star}\right)^{2}}{\left(J_{{IPP}}^{{\max}}-J_{IPP}^{\star}\right)^{2}}\\ &&+C_{N}\frac{\left(J_{N}(p)-J_{N}^{\star}\right)^{2}}{\left(J_{N}^{{\max}}-J_{N}^{\star}\right)^{2}} \end{array} $$

with well balanced weights $(C_{IP},C_{IPP},C_{N})\in \left \{\left (\frac {1}{3},\frac {1}{3},\frac {1}{3}\right),\left (\frac {1}{2},0,\frac {1}{2}\right)\right \}$ and *p*∈ defined in (??). For *p*=(*p*_*i*_)_*i*=1,…,9_∈, each *p*_*i*_ can vary within an interval whose bounds are constrained

- either a priori by natural considerations:
  - for instance *γ*(*t*) is a proportion, so that *γ*_0_,*γ*_1_∈[0,1].
- or a priori from modeling principles
  - The slopes of the sigmoid functions modeling temporal variations in *γ*(*t*) and *F*_*AP*_(*t*) are lower than 10. Such a value ensures that the rise and decay phases will last at least one day in the case when the asymptotes (*γ*_0_ and *γ*_1_) take the extremal values (0 and 1).
  - Also, the rise (resp. decay) in *F*_*AP*_ has to happen before (resp. after) the time when the maximal IP number is observed.
- or *a posteriori*
  - Bounds on *K*_*AP*_ are in order to remain inactive at the minimum.

In (27) the three elementary criteria measure the fit with each the three datasets

28 $$\begin{array}{@{}rcl@{}}J_{IP}(p)&=&\sum_{i=1}^{N_{exp}} w_{i}^{IP}\left(\overline{{IP}}(t_{i},p)-{IP}_{i}^{exp}\right)^{2}, \end{array} $$

29 $$\begin{array}{@{}rcl@{}} J_{{IPP}}(p)&=&\sum_{i=1}^{N_{exp}} w_{i}^{IPP}\left(\overline{{IPP}}(t_{i},p)-{IPP}_{i}^{exp}\right)^{2}, \end{array} $$

30 $$\begin{array}{@{}rcl@{}} J_{N}(p)&=&\sum_{i=1}^{N_{exp}} w_{i}^{N}\left(N(t_{i},p)-N_{i}^{exp}\right)^2. \end{array} $$

In each elementary criterion (28-30) entering the fit function (27), *IP*^*exp*^(*t*), *IPP*^*exp*^(*t*) and *N*^*exp*^(*t*) denote the averaged experimental values (average of individual measures for a given time point and a given genotype (WT or KO), see Additional file 3). The coefficients $w_{i}^{IP}$, $w_{i}^{IPP}$, and $w_{i}^{N}$ are not based on the empirical statistical variance, due to the low number of replicates, but they account for the variability in the experimental sampling times (some time points are one day apart, while others are half a day apart). $\overline {IP}(t,p)$ (respectively *N*(*t*,*p*), $\overline {IPP}(t,p)$) is the corresponding model output computed using the values of parameter vector *p* in Eqs. (1-8). The normalization of *J*_*IP*_(*p*), *J*_*IPP*_(*p*) and *J*_*N*_(*p*) in (27) uses the extremum values $J_{IP}^{\star }$ and $J_{IP}^{{\max }}$ (respectively $J_{IPP}^{\star }$, $J_{N}^{\star }$ and $J_{IPP}^{{\max }}$, $J_{N}^{{\max }}$) obtained by minimization of *J*_*IP*_(*p*) (respectively *J*_*IPP*_(*p*), *J*_*N*_(*p*)) as explained in more details in Additional file 4. An interesting feature of this normalized multi objective function is that it enables us to compare the results of optimization procedures performed on different datasets (control and mutant), as it ensures that *J*(*p*) varies between 0 and 1.

### Sensitivity analysis

In order to investigate the robustness of our calibration we perform a sensitivity analysis (detailed in Additional file 5) similar to that performed in [25, 55]. We study in turn the individual influences of the nine parameters over the fit function, around the parameter values of scenario 3. The optimal value of each parameter $p^{\star }_{j}$ is changed in turn in a ±10*%* range, while the other paramaters are left unchanged.

We compute the normalized sensitivity as done in [25]

$$\begin{array}{@{}rcl@{}} S(p_j/p_{j}^{\star})=\frac{J(p)-J(p^{\star})}{J(p^{\star})}. \end{array} $$

The behavior of *S* shows that the minimum found by CMAES [56] is very robust since all curves remain monotonous in the ±10*%* parameter range on each side of the optimum. The effects of the different parameters are quite contrasted. The fit function is sensitive mainly to the parameters delimiting the neurogenesis period, *t*_−_ and *t*_+_, and to a lesser extent to *t*_*γ*_, *γ*_0_, and *γ*_1_. The slope parameters *s*_+_, *s*_−_, and *s*_*γ*_ have the lowest influence.

We also perform an analysis of the sensitivity to noise in the data. We generate synthetic noisy datasets by adding normally distributed random noise ${\mathcal {N}}(0,\sigma)$ to the deterministic outputs, and we compute the statistics of the parameters identified on single noisy datasets. As a whole, all parameters, except the slope parameters, are well estimated in the case of low noise (with an error less than 1% for *K*_*AP*_, *t*_+_, *t*_−_, and *t*_*γ*_, 1.4*%* for *γ*_0_ and 3.6*%* for *γ*_1_).

As a result of this study, we conclude that the CMAES stochastic method, which overcomes the problem of sensitivity to the initial parameter guess, is more appropriate than a descent method.

### Computation of the number of double labeled cells in s phase-labeling experiments

The approximated number of double labeled cells, mimicking the experimental result and entering Eq. (25), can be computed from extracting the contribution of Brdu labeled IPP cells to the number of IPN in S phase at time *t*_2_,

31 $$ \overline{IPN}_{S}(t_{2})= \int_{T^{IPN}_{G1}}^{T^{IPN}_{G1}+T^{IPN}_{S}} IPN(t_{2},a)da \,.   $$

According to the specific choice of *t*_2_−*t*_1_, this quantity can be a mixture of:

1. Former IPP cells that were in S phase at *t*_1_, corresponding to cells already labeled with BrdU;
2. Former IPP cells that were outside S phase at *t*_1_, i.e. that had either overcome S phase (and were in M or G2 phase) or were still in G1 phase;
3. Former AP cells that divided within the [*t*_1_,*t*_2_] time interval and have reached phase S since then.

To stick to the experimental values and select only BrdU labeled cells within this mixture, we have to get of rid of cells in case 2 or 3.

The common point between the three cell sources is that cells have entered a new IPN cell cycle in the interval $\tau \in \left [t_{2}-T_{G1}^{IPN}-T_{S}^{IPN},t_{2}-T_{G1}^{IPN}\right ]$, with the lower bound corresponding to cells at the very end of S phase, and the upper bound to cells at the very beginning of phase S. An alternative way to compute $\overline {IPN}_{S}(t_{2})$ in Eq. (31) is thus to integrate boundary condition (4) in time along this interval:

32 $$ {}{\begin{aligned} \overline{IPN}_{S}(t_{2})= \int_{t_{2}-T^{IPN}_{S}-T^{IPN}_{G1}}^{t_{2}-T^{IPN}_{G1}}{\left\{(1-\gamma(\tau))\beta(\tau)F_{AP}(\tau)+2IPP\left(\tau,T_{C}^{IPP}\right)\right\}d\tau} \,. \end{aligned}}  $$

To extract the contribution of the proper cells (case 1) from (32), we first get rid of the contribution of AP cells, which is straightforward.

**Fig. 8:**
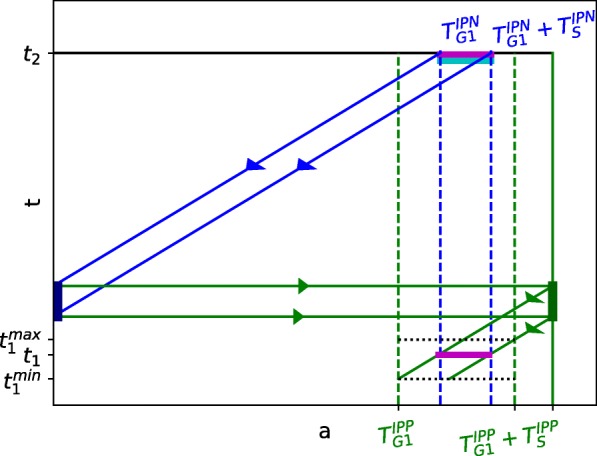
Sketch of the retrotracing of IPN S-labeled cells at *t*_2_ back to IPP S phase at *t*_1_. Double labeled cells (magenta-cyan horizontal segment), whose age at *t*_2_ belongs to $\left [T^{IPN}_{G1}, T^{IPN}_{G1}+T^{IPN}_{S}\right ]$, have initiated an IPN cycle in the time interval $\left [t_{2}-\left (T^{IPN}_{G1}+T^{IPN}_{S}\right),t_{2}-T^{IPN}_{G1}\right ]$ (navy vertical segment) instantaneously after exiting mitosis of a former IPP cycle, when they had reached age $T^{IPP}_{C}$ (darkgreen vertical segment). In addition, they had to be in S phase of the IPP cycle at *t*_1_, with their age belonging to $\left [T^{IPP}_{G1}, T^{IPP}_{G1}+T^{IPP}_{S}\right ]$. Production of additional cells through division is not explicitly figured here

For IPPs, things are a little more tricky. We take advantage of the formulation (26) of the solutions as characteristic curves to follow IPN cells labeled at *t*_2_ backwards in time (see the sketch of the computation on Fig. 8). At any time *τ* in Eq. (32), IPPs contributing to $\overline {IPN}_{S}(t_{2})$ are aged $T_{C}^{IPP}$, meaning that they were aged $T_{C}^{IPP}\,-\,(\tau \,-\,t_{1})$ at time *t*_1_. Hence we should keep only those cells whose age $T_{C}^{IPP}\,-\,(\tau \,-\,t_{1})$ at time *t*_1_ is compatible with their belonging to phase S at time, i.e. :

33 $$\begin{array}{@{}rcl@{}} && T_{G1}^{IPP}\leq T_{C}^{IPP}-(\tau-t_1) \leq T_{G1}^{IPP}+T_{S}^{IPP} \,, \\ && t_1+ T_{C}^{IPP}-T_{G1}^{IPP}-T_{S}^{IPP}\leq \tau \leq t_1+ T_{C}^{IPP}-T_{G1}^{IPP} \,.  \end{array} $$

Hence, the estimated number of double labeled cells at *t*_2_ is given by

34 $$ {}{\begin{aligned} \overline{IPN}_{S}^{\star}(t_{2})= \int_{\max\left(t_{2}-T^{IPN}_{S}-T^{IPN}_{G1}; \, t_{1}+ T^{IPP}_{C} - T^{IPP}_{G1}-T^{IPP}_{S}\right)}^{\min\left(t_{2}-T^{IPN}_{G1}; \, t_{1}+T^{IPP}_{C} -T^{IPP}_{G1}\right)}{2IPP\left(\tau,T_{C}^{IPP}\right)d\tau} \,, \end{aligned}}  $$

or equivalently

$${}{\begin{aligned} \overline{IPN}_{S}^{\star}(t_{2})= \int_{\max\left(t_{2}-T^{IPN}_{S}-T^{IPN}_{G1}; \, t_{1}+T^{IPP}_{G2}+T^{IPP}_{M}\right)}^{\min\left(t_{2}-T^{IPN}_{G1}; \, t_{1}+T^{IPP}_{G2}+T^{IPP}_{M}+T^{IPP}_{S}\right)}{2IPP\left(\tau,T_{C}^{IPP}\right)d\tau} \,. \end{aligned}} $$

Note that, since $T_{C}^{IPP}-(\tau -t_{1})$ can be rewritten as $T_{C}^{IPP}-[(t_{2}-t_{1})-(t_{2}-\tau)]$ condition (33) amounts to:

$${}{\begin{aligned} T_{C}^{IPP}-\left(T_{G1}^{IPP}+T_{S}^{IPP}\right) \leq &(t_{2}-t_{1})- (t_{2}-\tau)\leq T_{C}^{IPP}-T_{G1}^{IPP}\\ T^{IPP}_{G2}+T^{IPP}_{M} \leq &(t_{2}-t_{1})- (t_{2}-\tau)\leq T^{IPP}_{G2}+T^{IPP}_{M}+T^{IPP}_{S}. \end{aligned}} $$

In the case when $\tau =t_{2}-T^{IPN}_{G1}-T^{IPN}_{S}$, then

$$\begin{array}{*{20}l}T^{IPP}_{G2}&+T^{IPP}_{M}+ T^{IPN}_{G1}+T^{IPN}_{S}\leq (t_{2}-t_{1})\leq T^{IPP}_{G2}\\&+T^{IPP}_{M}+T^{IPN}_{G1}+T^{IPN}_{S}+T^{IPP}_{S}.\end{array} $$

In the case when $\tau =t_{2}-T^{IPN}_{G1}$, then

$$\begin{array}{*{20}l}T^{IPP}_{G2}&+T^{IPP}_{M}+ T^{IPN}_{G1}\leq (t_{2}-t_{1})\leq T^{IPP}_{G2}+T^{IPP}_{M}\\&+T^{IPN}_{G1}+T^{IPP}_{S}.\end{array} $$

Hence, to make sure to detect at least some of the cells labeled by BrdU at *t*_1_ (hence to get less than 100% false positive cells), the delay *t*_2_−*t*_1_ has to be bounded by

35 $$ {}{\begin{aligned} T^{IPP}_{G2}+T^{IPP}_{M}+ T^{IPN}_{G1}\leq (t_{2}-t_{1})\leq T^{IPP}_{G2}+T^{IPP}_{M}+T^{IPN}_{G1}+T^{IPN}_{S}+T^{IPP}_{S}. \end{aligned}}  $$

## Discussion

We have undertaken an interdisciplinary approach to study the dynamics of neurogenesis in the developing mammalian cerebral cortex. Our work combines an experimental quantitative analysis of cell populations along the neurogenesis process in the mouse cortex, with mathematical modeling, numerical simulation and parameter optimization. The main results of this work are the following:

- We have built a compartmental model, which not only accounts for the temporal changes in cell numbers, but also embeds a multiscale description of the IP dynamics. IP cells can be tracked along the cell cycle as they progress into the subsequent cell phases until division, and quantitative information is available at the same time on several scales.
- Our formalism provides one with a framework to study how progenitor numbers, division modes and proliferation rates interact to control quantitatively and temporally the neuronal output.
- We successfully adjusted the model parameters to our experimental data on neurogenesis in the developing murine cerebral cortex in control conditions, which has enabled us to predict progenitor dynamics in a mutant context.
- We show how to compute cell kinetics indexes from the model outputs and to optimize experimental protocols intending to discriminate the different progenitor types.
- The model can be used in a straightforward manner to study other mouse mutants with cortical neurogenesis defects in a dedicated simulation environment (CEMONE).

In our approach, the model assumptions are based on state-of-the-art knowledge on cortex cell dynamics during development [6, 46] as far as (i) identification of cell types (distinction between APs and IPs, and further between IPPs and IPNs), (ii) cell flows between cell populations (neurogenic versus IPgenic division in APs and IPs), as well as between-type differences in cell cycle and cell phase durations. We mainly focused on IP cell dynamics, without representing explicitly the asymmetric divisions of APs. Similarly, to describe cell lineage specification within the inner cell mass, the authors in [57] did not account as such for the cell dynamics in the trophectoderm, and only considered the contribution of asymmetric divisions to the increase in the inner cell pool. In addition to giving a good fit with experimental IP and neuron cell numbers, the model output also predicts trends in the neurogenic fraction that are consistent with the detailed results provided in [46]. These predictions corroborate our choices of formulation for functions *β*(*t*) and *γ*(*t*). To check the interest of dealing with time-dependent rates versus constant ones, we have performed a series of preliminary trials with either *β*(*t*) or *γ*(*t*) constant, or both. From these optimization results, it appeared that there was a real gain in keeping *γ*(*t*) time-dependent (see for instance the comparison between the 3 control scenarios in Fig. 5). In contrast, rate *β*(*t*) could be kept constant to a very low value, and even set to 1. Such a conclusion was not so obvious at the time we obtained it, yet it has been since then supported by a recent thorough study [58], which shows that direct neurogenesis is very low in the neocortex. More specifically, in our modeling framework, much of the complexity is captured by the repartition of IPs into neurogenic and IPgenic cells and by the consequences resulting from the differences in their transit times along the whole cell cycle duration and individual cell cycle phases. In particular, the difference in phase S duration, revealed by [6], have important consequences on the temporal pattern of the labeling index, which should be taken into account to interpret cell kinetics studies, including the most recent ones [59]. IPs have not been reported to be subject to apoptosis, neither are they susceptible to undergo a glial transition in a significant way, so that they participate entirely in the neuronal output. In addition, we have not seen any sign of IP apoptosis in our data. We settled the model assumptions accordingly. Up to now, IPgenic IPs can be discriminated from neurogenic IPs in a direct way only in the *T*is21-GFP transgenic mouse line [7]. The Expression of *T*is21 coupled to *G*FP allows one to investigate the change in the neurogenic fraction within the IP population, as performed in depth in [46]. To our knowledge, no method enabling the direct lineage tracing of IPgenic cells through subsequent cell cycles is yet available. IP dependent lineages can be identified thanks to dedicated protocols which can evidence the passage through a Tbr2+, IP state [9], yet cannot determine the number of consecutive cell generations in the IP state. The question of the maximal number of cell cycles performed by this cell type remains open. In particular, it is not known whether a significant proportion of IPs can undergo more than two cell cycles, even if this hypothesis is raised from some experimental observations of increased IP renewal [45], that motivated our in silico study on double phase S labeling. Interpretation of these results are nevertheless tricky, and further complicated by the fact that Tbr2 labeling is impermanent.

In our model, we considered that IP cells can progress along either 1 or 2 cell cycles before engendering post-mitotic neurons, even if it would be very easy to allow IPgenic cells to re-enter an IPgenic cell cycle (at *IPP*(*t*,*a*=0)) with a slight modification of boundary condition (3). There are multiple reasons underlying this choice. First, as stated just above, from an estimation viewpoint the return rate in IPgenic cell cycles would add a nonidentifiable parameter in the model. Second, to a large extent, additional cell cycle turnover is not needed to produce high levels of IPs (and IPPs), which can be achieved by tuning *γ*(*t*) accordingly. Third, adding a cell cycle in the sequence lengthens the period during which IP cells remain present. Lengthening this period would not be have been particularly consistent with the sharp decrease observed in our experimental cell numbers after the peak value. In contrast, in [16], the possibility of progressing through a third cycle as IP has been considered to explain the phenotype of compensation for an abundant apoptosis in the post-mitotic compartment, observed in their *D*bx1 mutant (note that the model assumptions are similar to ours in the control case, with at most two cycles for IPs and no apoptosis in either IPs or neurons during the neurogenesis period).

We used the model to study cell dynamics in a pathological context, the *F*tm/Rpgrip1l mouse mutant, a model of ciliopathies, which shows a complex and time-dependent neurogenesis phenotype. At early stages of corticogenesis, we observed reduced numbers of IPs and neurons. However, this does not reflect a constant impairment in neurogenesis, since at later stages, neurons form and IP numbers are transiently increased. Can this complex phenotype find explanation in terms of population dynamics? Two main (non exclusive) mechanisms could explain the peak of IPs that occurs at E15.5-E16.5. IPs could have, at least transiently, an increased proliferative potential, which would translate in the model into the temporal pattern of *γ*(*t*). Alternatively, IPs could be produced in higher number from APs, which would translate into the temporal pattern of *F*_*AP*_(*t*). The result of the optimization procedure for the mutant favors the prominence of the second mechanism: *F*_*AP*_(*t*) shows a skewed pattern with a quite narrow peak at E15.5-E16.5, whereas both the instantaneous and cumulated values of *γ*(*t*) remain below the control in the mutant (Fig. 6). Moreover, for a strictly identical AP input, increasing IPP numbers through *γ*(*t*) leads to a higher *r*_*AP*_(*t*), hence a higher number of neurons at the end of corticogenesis, which is not observed in the *F*tm mutant (Fig. 3). Moreover, the earlier and sharper decay observed in the mutant *F*_*AP*_(*t*) is expected to reflect a premature end of neurogenesis, which fits with our experimental data (Supplementary Table A2.1). The transient increase in neurogenesis at the peak could be a compensatory mechanism, caused for instance by a higher packing of APs at the ventricular surface or by a reduced number of neurons in the cortical plate, inducing a change in AP division orientation, which is known to contribute to the fate of AP progeny (reviewed in ([60]).

Alternatively, this transient peak could be a secondary consequence of cilia loss, independent of the early delay in neurogenesis. Interestingly, the conditional inactivation of *I*ft88 and *K*if3a, two genes essential for ciliogenesis, leads to a modest increase in the IP production in the absence of neurogenesis delay ([61]), suggesting that these two events could be independent of each other.

In the cerebral cortex, different types of neurons are produced in a precise temporal order and form six major neuronal layers with distinct properties, connectivity and molecular signatures, that can be fully identified after birth [1–3]. Our model could be adapted to study the temporal dynamics of neuronal subtype production ; one almost immediate step forward in this direction would consist in assigning a layer to neurons according to their birth date. The results of the optimization procedure has highlighted the interest of choosing a time varying expression for *γ*(*t*), the parameter tuning the proportion of AP-derived IPgenic (versus neurogenic) newborn IPs. In population dynamics, time-varying transfer rates are often the signature of the existence of feedback controls between cell populations. The role of feedback regulations of the progression through (generic) cell lineages during morphogenesis (hence in a different framework than tissue homeostasis) has been thoroughly investigated from an interesting theoretical biology viewpoint in [62]. An extension of our work could consist in embedding our model, dealing with specific cell types, within a similar framework. Yet, this would require accurate functional knowledge on the dynamic interactions between cell types, which are not yet available, even if some pieces of the puzzle could be used right now, such as for instance the impact of immature neurons on AP fate [59] (hence on *F*_*AP*_ in our framework). Also, feedback interactions between cell populations is only one of the source of time-dependency. The sequence of proliferation and differentiation of neural progenitors in the cortex is subject to numerous intrinsic and extrinsic dynamical processes [42], among which (i) cell-autonomous programming along successive cell generations (sequential genetic expression of molecular markers associated with an increasing differentiation status) (reviewed in [63]), (ii) spatio-temporal gradients of morphogens originating from organizing centers in the brain (reviewed in [64]) and (iii) transient cell-cell interactions between the different cell types, including possible feedback from post-mitotic cells onto progenitor cells (reviewed in [26]).

As another, alternative model extension, we could consider describing the dynamics of AP cells with the same granularity as we do for IP cells, which would for instance allow us to tackle the question of the possible relations between the lengthening of phase G1, and progenitor fating towards one type of division or another [65]. Yet, at this stage, due to the difficulty of retrieving and exploiting quantitative data on AP cell numbers, such an approach would be based only on direct numerical simulations, and would thus remain rather theoretical.

## Conclusions

In this study, we have designed a multiscale mathematical model for neural progenitor dynamics in the mouse cerebral cortex. We have fitted the model outputs to experimental cell numbers observed from mouse embryos at different stages of cortical development. The parameter calibration is based on a multi-objective optimization function computed from both IPs and neurons. The model also gives access on cell kinetics markers, such as the mitotic and S phase indexes, and derived information as the fraction of neurogenic mitoses or monitoring of S-labeled cells along successive cell cycles. The fitted parameters obtained for a mouse mutant for *F*tm/Rpgrip1l are quite different from the control case. They reveal a shortening of the neurogenic period associated with an increased influx of newborn IPs from apical progenitors at mid-neurogenesis. Beyond our specific interest for *F*tm/Rpgrip1l, one could easily use our framework to study other experimental situations in rodents. The quantitative effect of changing some parameter values can be tested and visualized in a user-friendly and interactive manner in the CEMONE environment (ipython notebook).

## Additional files

Additional file 1 Computation of labeling and mitotic indexes in the constant *γ* case. (PDF 482 kb)

Additional file 2 Histological and morphometric criteria used for staging control brain sections. (PDF 1715 kb)

Additional file 3 Cell counts. Values in columns D, E and H correspond to the dots in Fig. 3g, f and h. Lines labeled with Ctrl correspond to wild type (WT) in blue and *F*tm-/- to (KO) mutant in red. Points at a given age correspond to different embryos, from up to two different litters. (XLSX 14 kb)

Additional file 4 Weights and parameter bounds in the multi objective function. (PDF 751 kb)

Additional file 5 Sensitivity analysis. (PDF 508 kb)

Additional file 6 Calibration using mitotic index data. (PDF 322 kb)

Additional file 7 Identifiability. (PDF 240 kb)

## Abbreviations

AP: Apical progenitor
BP: Basal progenitor
BrdU: Bromodeoxyuridine
CEMONE: CEll based MOdel of NEurogenesis
CP: Cortical plate
Ctip2: C2H2-type zinc finger protein 2 / Bcl11b
Ctrl: Control
DAPI: 4?,6-diamidino-2-phenylindole
Dbx1: Developing Brain homeobox 1
DNA: Deoxyribonucleic acid
E: Embryonic day
Ftm: Fantom
INM: Interkinetic nuclear migration
IP: Intermediate progenitor
IPP: Proliferative intermediate progenitor
IPN: Neurogenic intermediate progenitor
IZ: Intermediate zone
KO: Knock out mutant
LI: Labeling index
MI: Mitotic index
N: Neuron
OCT: Optimum cutting temperature
Pax6: Paired box protein
PBS: Phosphate buffered saline
PDE: Partial differential equation
PFA: Paraformaldehyde
PH3: Phosphohistone H3
Rpgrip1l: Retinitis pigmentosum GTPase regulator protein 1 like
Satb2: Special AT-Rich sequence-binding protein 2
SVZ: Subventricular zone
Tbr2: Eomesodermin also known as T-box brain protein 2
th: Thickness
VZ: Ventricular zone
w: width
WT: Wild type
