# Supplementary material for: A multiscale mathematical model of cell dynamics during neurogenesis in the mouse cerebral cortex
Source: BMC Bioinformatics. 2019 Sep 14;20:470. doi: 10.1186/s12859-019-3018-8 (PMC6744691; doi:10.1186/s12859-019-3018-8)
Supplement: Supplementary file 1 — Computation of labeling and mitotic indexes in the constant γ case. (PDF 482 kb) [file 12859_2019_3018_MOESM1_ESM.pdf]

Additional file 1. Computation of labeling and mitotic indexes in the constant γ case

In the case when the division rate γ is constant the solution of equations (1-4) can be solved explicitly using the method of characteristics

$$\begin{aligned}
IPP(t,a) &= \gamma F_{AP}(t-a) \\
IPN(t,a) &= (1-\gamma)F_{AP}(t-a) + 2\gamma IPP(t-a,T_c) \\
&= (1-\gamma)F_{AP}(t-a) + 2\gamma F_{AP}(t-T_c-a).
\end{aligned}$$

Therefore, for $t > 2T_c$, the total number of IPs is

$$\begin{aligned}
\overline{IP}(t) &= \overline{IP}^{IPP}(t) + \overline{IP}^{IPN}(t) \\
&= \int_0^{T_c} IPP(t,a)da + \int_0^{T_c} IPN(t,a)da \\
&= \int_0^{T_c} \gamma F_{AP}(t-a)da \\
&\quad + \int_0^{T_c} \left((1-\gamma)F_{AP}(t-a) + 2\gamma F_{AP}(t-T_c-a)\right) da \\
&= \int_{t-T_c}^{t} F_{AP}(s)ds \\
&\quad + 2\gamma \int_{t-2T_c}^{t-T_c} F_{AP}(s)ds.
\end{aligned}$$

Similarly, the total number of mitotic IPs is

$$\begin{aligned}
\overline{IP}_M(t) &= \overline{IP}_M^{IPP}(t) + \overline{IP}_M^{IPN}(t) \\
&= \int_{T_c-T_M}^{T_c} IPP(t,a)da + \int_{T_c-T_M}^{T_c} IPN(t,a)da \\
&= \int_{T_c-T_M}^{T_c} \gamma F_{AP}(t-a)da \\
&\quad + \int_{T_c-T_M}^{T_c} \left((1-\gamma)F_{AP}(t-a) + 2\gamma F_{AP}(t-T_c-a)\right) da \\
&= \int_{t-T_c}^{t-T_c+T_M} F_{AP}(s)ds \\
&\quad + 2\gamma \int_{t-2T_c}^{t-2T_c+T_M} F_{AP}(s)ds
\end{aligned}$$

and the total number of IPs in S phase is

$$
\begin{aligned}
\overline{IP}_S(t) &= \overline{IP}_S^{IPP}(t) + \overline{IP}_S^{IPN}(t) \\
&= \int_{T_{G1}}^{T_{G1}+T_S} IPP(t,a)da + \int_{T_{G1}}^{T_{G1}+T_S} IPN(t,a)da \\
&= \int_{T_{G1}}^{T_{G1}+T_S} \gamma F_{AP}(t-a)da \\
&\quad + \int_{T_{G1}}^{T_{G1}+T_S} \left((1-\gamma)F_{AP}(t-a) + 2\gamma F_{AP}(t-T_c-a)\right) da \\
&= \int_{t-T_{G1}-T_S}^{t-T_{G1}} F_{AP}(s)ds \\
&\quad +2\gamma \int_{t-T_c-T_{G1}-T_S}^{t-T_c-T_{G1}} F_{AP}(s)ds
\end{aligned}
$$

Combining partial and total cell counts we obtain the labeling index

$$
LI(t) = \frac{\displaystyle\int_{t-T_{G1}-T_S}^{t-T_{G1}} F_{AP}(s)ds + 2\gamma \int_{t-T_c-T_{G1}-T_S}^{t-T_c-T_{G1}} F_{AP}(s)ds}{\displaystyle\int_{t-T_c}^{t} F_{AP}(s)ds + 2\gamma \int_{t-2T_c}^{t-T_c} F_{AP}(s)ds}
$$

and the mitotic index

$$
MI(t) = \frac{\displaystyle\int_{t-T_c}^{t-T_c+T_M} F_{AP}(s)ds + 2\gamma \int_{t-2T_c}^{t-2T_c+T_M} F_{AP}(s)ds}{\displaystyle\int_{t-T_c}^{t} F_{AP}(s)ds + 2\gamma \int_{t-2T_c}^{t-T_c} F_{AP}(s)ds}.
$$

The variation rate in time and in γ is not obvious since both numerator and denominator are positive and increasing functions of γ.

Simpler expressions can be derived for steady state AP input flux $F_{AP}(t) = K_{AP}$, even when IPPs and IPNs have different phases and cell cycle durations. In that case, for $t > T_c^{IPP} + T_c^{IPN}$ (both IP compartments full) we have

$$
\begin{aligned}
IPP(t,a) &= \gamma K_{AP} \\
IPN(t,a) &= (1-\gamma)K_{AP} + 2\gamma K_{AP} = (1+\gamma)K_{AP}
\end{aligned}
$$

Therefore, the total number of IPs is constant in time and equal to

$$
\begin{aligned}
\overline{IP} &= \overline{IP}^{IPP}(t) + \overline{IP}^{IPN}(t) \\
&= \int_0^{T_c^{IPP}} IPP(t,a)da + \int_0^{T_c^{IPN}} IPN(t,a)da \\
&= \gamma K_{AP} T_c^{IPP} + (1+\gamma)K_{AP} T_c^{IPN} \\
&= K_{AP}(\gamma T_c^{IPP} + (1+\gamma)T_c^{IPN}).
\end{aligned}
$$

Similarly, the total number of mitotic IPs is

$$\begin{aligned}
\overline{IP}_M &= \overline{IP}_M^{IPP}(t) + \overline{IP}_M^{IPN}(t) \\
&= \int_{T_c^{IPP}-T_M^{IPP}}^{T_c} IPP(t,a)da + \int_{T_c^{IPN}-T_M^{IPN}}^{T_c} IPN(t,a)da \\
&= T_M^{IPP}\gamma K_{AP} + T_M^{IPN}(1+\gamma)K_{AP} \\
&= K_{AP}(\gamma T_M^{IPP} + (1+\gamma)T_M^{IPN})
\end{aligned}$$

and the total number of IPs in S phase is

$$\begin{aligned}
\overline{IP}_S &= \overline{IP}_S^{IPP}(t) + \overline{IP}_S^{IPN}(t) \\
&= \int_{T_{G1}^{IPP}}^{T_{G1^{IPP}}+T_S^{IPP}} IPP(t,a)da + \int_{T_{G1}^{IPN}}^{T_{G1}^{IPN}+T_S^{IPN}} IPN(t,a)da \\
&= K_{AP}(\gamma T_S^{IPP} + (1+\gamma)T_S^{IPN}),
\end{aligned}$$

from which we derive the index expressions

$$LI = \frac{\gamma T_S^{IPP} + (1+\gamma)T_S^{IPN}}{\gamma T_c^{IPP} + (1+\gamma)T_c^{IPN}} \tag{A1-1}$$

$$MI = \frac{\gamma T_M^{IPP} + (1+\gamma)T_M^{IPN}}{\gamma T_c^{IPP} + (1+\gamma)T_c^{IPN}}. \tag{A1-2}$$

In the case when the duration and phase-structuring of IPP and IPN cell cycles are set to the same values, (A1-1-A1-2) boil down to the simplest relation corresponding to a stationary population with uniform distribution in age:

$$LI = \frac{T_S^{IPN}}{T_c^{IPN}}, \qquad MI = \frac{T_M^{IPN}}{T_c^{IPN}}.$$

Panels A and B of Fig. A1-1 illustrate the deviation from this idealized situation induced by the time-varying character of F_{AP}. The plateau phase ($\approx$10.68 for LI and ≈ 1.9 for MI) in all 3 cases ($\gamma = 0$, 0.5 or 1) corresponds to the stationary configuration, during which F_{AP} is almost constant (see the blue line in panel B of Fig. 6.). The length of the plateau, hence the delay before reaching the stationary regime, is mainly tuned by the value of γ ; the plateau is reached earlier when there are only IPN cells ($\gamma = 0$). Apart from the plateau, the effect of either increasing or decreasing $F_{AP}(t)$ is clearly visible. Also, the indexes are only displayed in the time window E12-E16, during with the IPP and IPN cell cycles are filled of cells, and the index formulas are valid.

Panels D and E illustrate how the index patterns get complicated, and not intuitively predictable, in the realistic situation involving a time-varying $\gamma(t)$ and different durations of the cell cycle phases for IPP and IPN. Not only the indexes do not reach any stationary regime, but they are also non monotonic and their ordering can change with time between the scenarios (for each scenario,

the constant values computed from equations (A1-1-A1-2) are shown as dashed lines).
In both the simplified and realistic configuration, the corresponding neurogenic fraction (proportion of IPN mitoses amongst the whole IP mitoses) are shown on the rightmost panels (C and F).

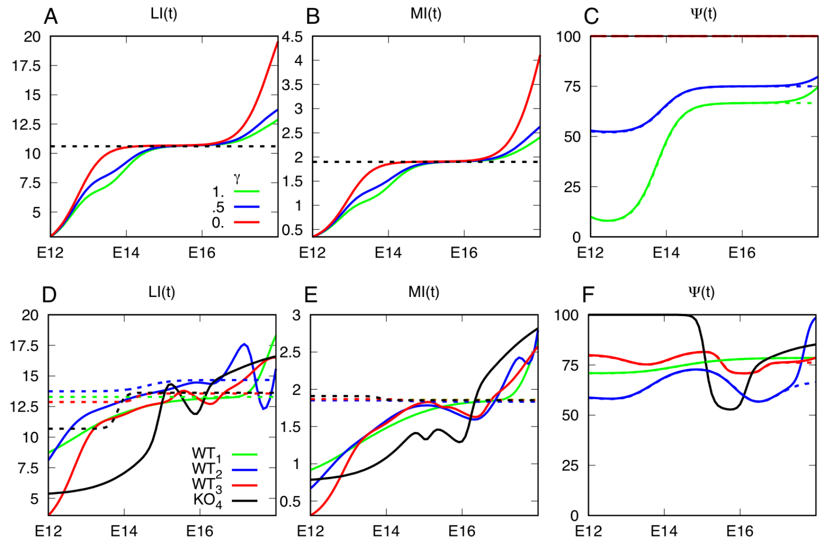

Figure A1-1: Panels A, B and C. Cell kinetics indexes for a simplified parameter set (F_{AP}, from scenario 3 of Table 4, $\beta = 1$, and identical cell cycle phases for both IPPs and IPNs, second line in 3). We keep $\gamma(t) = cte$ with three possible values 0 (in red), 0.5 (in blue) and 1 (in green). Panel A displays the labeling index (LI, in percent) for total IPs (13). Panel B displays the mitotic index (MI, in percent) for total IPs (14). Dashed lines indicate the theoretical index level with constant AP input flux. Panel C displays the ratio of neurogenic IP mitoses (in percent), $\psi(t)$, computed with (23) (solid lines), along with the estimate (24) (dashed lines). Panels D, E, and F : corresponding indexes for the 4 scenarios in Table 4; dashed curves indicate the theoretical index level with constant AP input flux and constant γ. At time t the displayed values correspond to $\gamma = \gamma(t)$ for each scenario.
