# Supplementary material for: A multiscale mathematical model of cell dynamics during neurogenesis in the mouse cerebral cortex
Source: BMC Bioinformatics. 2019 Sep 14;20:470. doi: 10.1186/s12859-019-3018-8 (PMC6744691; doi:10.1186/s12859-019-3018-8)
Supplement: Supplementary file 2 — Histological and morphometric criteria used for staging control brain sections. (PDF 1715 kb) [file 12859_2019_3018_MOESM2_ESM.pdf]

Additional file 2. Histological and morphometric criteria used for staging control brain sections

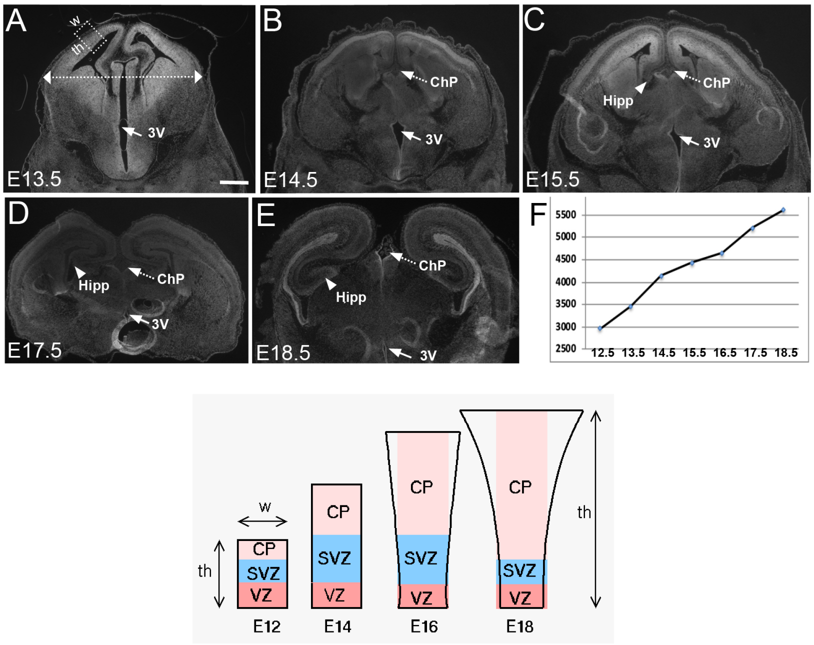

Figure A2-1: (A-E) Representative pictures of brain sections from control embryos used in this study. The dotted double arrow in A shows the width of the brain section. Full arrows point to the ventral part of the third ventricle (3V); dotted arrows point to the choroid plexus (ChP) present in the dorsal part of 3V from E14.5 onward; arrowheads point to the hippocampus (Hipp), showing its progressive folding. In panel A, the window used for the quantification is framed. w: width; th: thickness. F) Graph illustrating the linear progression of brain width with the embryonic stage, using reference sections taken from the Allen Atlas of the developing mouse brain (http://developingmouse.brain-map.org/static/atlas). G) 2D schematic representation of the effect of the deformation of the cortical plate on the experimental set-up.
