# Supplementary material for: A multiscale mathematical model of cell dynamics during neurogenesis in the mouse cerebral cortex
Source: BMC Bioinformatics. 2019 Sep 14;20:470. doi: 10.1186/s12859-019-3018-8 (PMC6744691; doi:10.1186/s12859-019-3018-8)
Supplement: Supplementary file 4 — Weights and parameter bounds in the multi objective function. (PDF 751 kb) [file 12859_2019_3018_MOESM4_ESM.pdf]

## Additional file 4. Weights in the multi objective function

In this paragraph we describe some technical issues in the minimization method used to calibrate the parameters of $\gamma$ and $F_{AP}$.

The coefficients $w_i^{IP}$, $w_i^{IPP}$, and $w_i^N$ weight the contribution of an individual experimental point in the fit function. They are not based on the empirical statistical variance, due to the low number of replicates, but they account for the variability in the experimental sampling times (some time points are one day apart, while others are half a day apart). They can be set either to 0 (to discard any obvious outliers, which is the case of the last two datapoints in neuron datasets), or in accordance to the experimental time sampling rate

$$\begin{aligned} w_i^X &= \frac{t_{i+1} - t_{i-1}}{2} \quad i = 2, \ldots, N_{exp} - 1 \\ w_1^X &= 1 \\ w_{N_{exp}}^X &= 1 \end{aligned}$$

The normalization of $J_{IP}(p)$, $J_{IPP}(p)$ and $J_N(p)$ in (27) uses the extremum values $J_{IP}^\star$ and $J_{IP}^{\max}$ (respectively $J_{IPP}^\star$, $J_N^\star$ and $J_{IPP}^{\max}$, $J_N^{\max}$) obtained by minimization of $J_{IP}(p)$ (respectively $J_{IPP}(p)$, $J_N(p)$). Using each dataset $X \in \{IP, IPP, N\}$ separately, we find an optimal parameter set $p_X^\star$ and optimal fit value

$$J_X^\star = J_X(p_X^\star). \tag{A1-3}$$

For each $p_X^\star$ obtained with a given dataset (WT/N, WT/IPP, or WT/IP, on one side, KO/N, KO/IPP, or KO/IP on the other side); we compute the a posteriori value of the fit function obtained with the two other datasets, to compute an approximation of $J_X^{\max}$

$$J_X^{\max} = \max_{Y \neq X} \left( J_X \left( p_Y^\star \right) \right).$$

An interesting feature of this normalized multi objective function is that it enables us to compare the results of optimization procedures performed on different datasets (control and mutant), as it ensures that $J(p)$ varies between 0 and 1.
The numerical values of $J_X^\star$ and $J_X^{\max}$ are gathered in Table A4-1 for the control and mutant datasets. The corresponding model inputs and outputs are displayed in Fig. A4-1. The important variability in the values of $J_{IP}$, $J_{IPP}$, and $J_N$ highlights the importance of performing a careful multicriterion calibration.

| dataset | $J_{IP}$ | $J_{IPP}$ | $J_N$ |
|---|---|---|---|
| WT/N | 406737 | 40847 | 14415 |
| WT/IPP | 169938 | 850 | 756363 |
| WT/IP | 3474 | 24676 | 1113922 |

| dataset | $J_{IP}$ | $J_{IPP}$ | $J_N$ |
|---|---|---|---|
| KO/N | 334449 | 204488 | 16261 |
| KO/IPP | 912652 | 1558 | 374392 |
| KO/IP | 3094 | 41526 | 378680 |

**Table A4-1** Values of the fit function for the control (left) and mutant (right) datasets. $J_X^{\max}$ is the max in each column (in red), $J_X^\star$ is the min on each line (in blue).

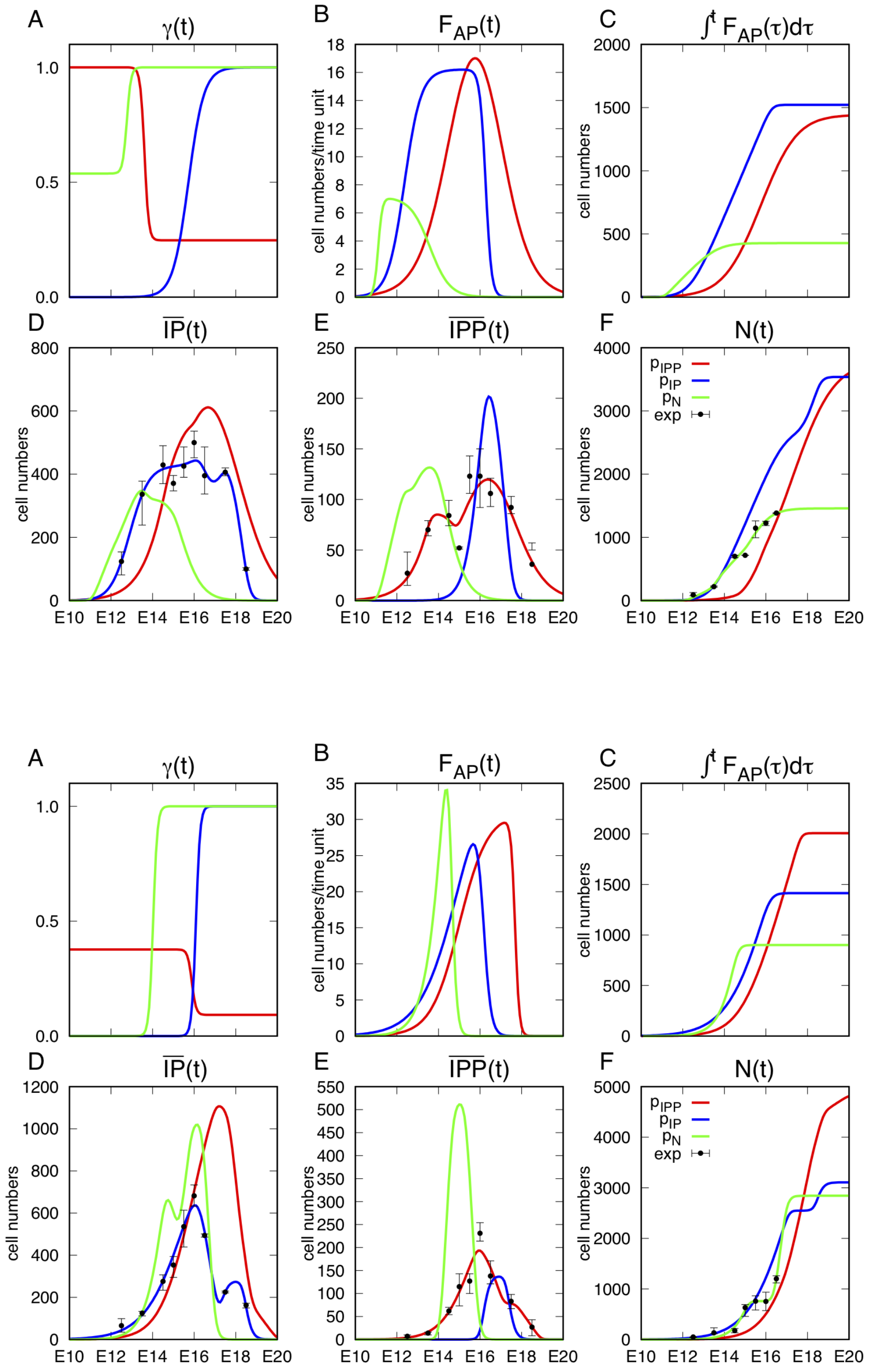


**Figure A4-1** Estimated changes in $F_{AP}(t)$ and $\gamma(t)$ obtained from optimizing the parameter values on the separate datasets (green curve : neuron dataset, blue curve: IP dataset, red curve: IPP dataset). Top panels: control, bottom panels: mutant
