# Supplementary material for: A multiscale mathematical model of cell dynamics during neurogenesis in the mouse cerebral cortex
Source: BMC Bioinformatics. 2019 Sep 14;20:470. doi: 10.1186/s12859-019-3018-8 (PMC6744691; doi:10.1186/s12859-019-3018-8)
Supplement: Supplementary file 5 — Sensitivity analysis. (PDF 508 kb) [file 12859_2019_3018_MOESM5_ESM.pdf]

Additional file 5. Sensitivity analysis

Fig. A5-1 illustrates the result of a sensitivity analysis performed with the param-

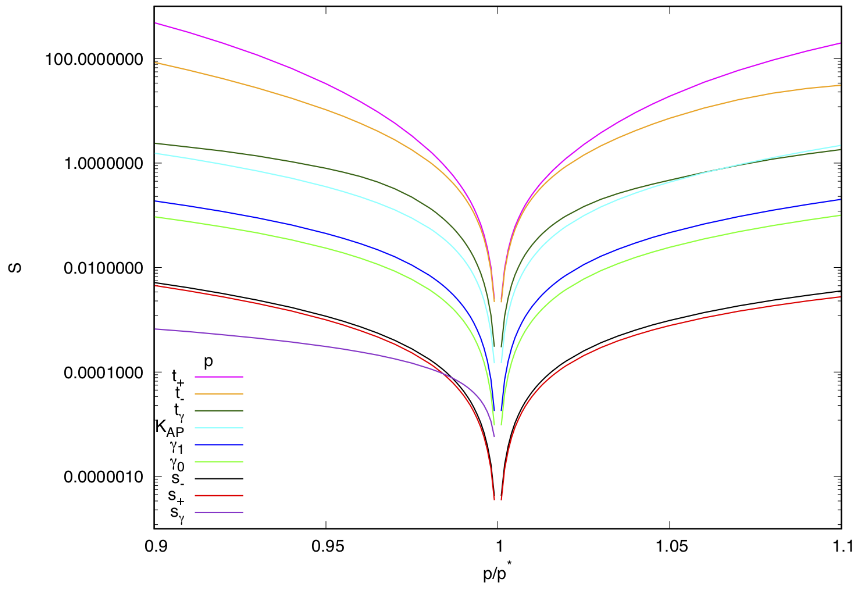

Figure A5-1 Sensitivity analysis for scenario 3 (column 3 of Table 4) in which parameters of $F_{AP}(t)$ ($K_{AP}, s_-, t_-, s_+, t_+$) and $\gamma(t)$ (γ_0, γ_1, t_γ, s_γ) have been estimated using all 3 (IP, IPP, and N) datasets. Each curve corresponds to the variation in criterion $J(p)$ for a single parameter, the values of the other eight parameters being left unchanged.

eter values corresponding to scenario 3 (cf column 3 of Table 4, as well as Table 1 for the definition of each parameter). The optimal value of each parameter $p_j^\star$ is changed in turn in a $\pm 10\%$ range, while the other paramaters are left unchanged. We compute the normalized sensitivity as done in [25]

$$S(p_j/p_j^\star) = \frac{J(p) - J(p^\star)}{J(p^\star)}.$$

The behavior of S shows that the minimum found by CMAES is very robust since all curves remain monotonous in the $\pm 10\%$ parameter range on each side of the optimum. The effects of the different parameters are quite contrasted. The fit function is sensitive mainly to the parameters delimiting the neurogenesis period, t_- and t_+, and to a lesser extent to t_γ, γ_0, and γ_1. The slope parameters s_+, s_-, and s_γ have the lowest influence. The scale of amplitudes observed in $S(p_j/p_j^\star)$ clearly suggests that a descent (gradient-like) method would get stuck in local minima and fail to find the optimal parameter values.

In order to investigate the robustness of the model with respect to the level of noise in the data we perform an in silico experiment .We compute the model outputs,

μ	σ	χ^2	p_{value}
14	69	1.55	0.45

Table A5-1 Statistics observed on 91 samples corresponding to distribution of residues with noised synthetic data using the parameter values of scenario 3

still with the parameter values of scenario 3. We generate random datasets

$$\{\overline{IP}(t_n), \overline{IPP}(t_n), N(t_n), \quad t_n = 10 + n/2,\ n = 0, \ldots, 19\}$$

by adding normally distributed random noise $\mathcal{N}(0, \sigma)$ to the deterministic outputs. We perform the calibration **separately** on each noised dataset (**hence through a single criterion approach without weighting the data points**) and compute the mean and standard deviation for each parameter. Table A5-1 displays the results for three different levels of noise $\sigma = 20$, 40 and 69, this latest value corresponding to the noise level estimated from our datasets. The results are consistent with the sensitivity analysis: the switch-like parameters (t_+, t_-, t_γ) only change a little with increasing noise level. In contrast, the slope parameters (s_+, s_- and s_γ) are sensitive to even small noise levels. The other parameters are not sensitive to low noise levels ($\sigma = 20$), yet their values depart a little from the expected values for rather high noise levels. As a whole, all parameters except the slope parameters, are well estimated in the case of low noise (with an error less than 1% for ${}_KAP$, t_+, t_-, and t_γ, 1.4% for γ_0 and 3.6% for γ_1).

Statistics on the parameters identified on randomized datasets

		Scenario 3	$\sigma = 20,\ \mu = 0$			$\sigma = 40,\ \mu = 0$			$\sigma = 69,\ \mu = 0$		
parameter name		p	$\langle p \rangle$	σ_p	$\frac{\langle p \rangle - p}{p}$	$\langle p \rangle$	σ_p	$\frac{\langle p \rangle - p}{p}$	$\langle p \rangle$	σ_p	$\frac{\langle p \rangle - p}{p}$
F_{AP}	K_{AP}	8.898	8.959	0.600	0.007	11.306	4.083	0.271	12.772	6.114	0.435
	s_+	3.168	3.777	2.914	0.192	6.379	4.115	1.014	5.871	4.158	0.853
	t_+	11.911	11.952	0.218	0.003	12.340	0.923	0.036	12.420	0.984	0.043
	s_-	3.123	4.183	2.471	0.339	5.683	4.100	0.820	5.893	4.162	0.887
	$t_- - t_+$	5.227	5.182	0.339	-0.009	4.397	1.085	-0.159	4.369	1.632	-0.164
γ	γ_0	0.280	0.284	0.125	0.014	0.351	0.267	0.251	0.389	0.317	0.388
	γ_1	0.456	0.472	0.071	0.036	0.587	0.280	0.288	0.583	0.346	0.279
	s_γ	10.000	8.262	2.904	-0.174	7.842	3.764	-0.216	9.007	2.545	-0.099
	t_γ	14.322	14.227	0.779	-0.007	14.826	1.262	0.035	14.523	1.514	0.014

Table A5-2 *The values of the paramers used to generate the model outputs are in column p. The mean values $\langle p \rangle$ and standard deviation σ_p are computed on at least 40 replicates.*
