# Supplementary material for: A multiscale mathematical model of cell dynamics during neurogenesis in the mouse cerebral cortex
Source: BMC Bioinformatics. 2019 Sep 14;20:470. doi: 10.1186/s12859-019-3018-8 (PMC6744691; doi:10.1186/s12859-019-3018-8)
Supplement: Supplementary file 6 — Calibration using mitotic index data. (PDF 322 kb) [file 12859_2019_3018_MOESM6_ESM.pdf]

Additional file 6. In silico calibration experiment using MI data

Data on cell kinetics indexes as MI or LI are mostly provided as snapshots, and are very rarely available as time series. Yet, merging macroscopic information on cell numbers, with mesoscopic information derived from cell kinetics indexes would be useful for giving access to more parameters (e.g the duration of cell cycle).
To substantiate this conjecture, we illustrate here numerically the gain provided by using a combination of cell numbers and MI datasets in the estimation procedure. We use a simplified estimation setup with $\gamma(t) = 0$, $\beta(t) = cte$, in which the model outputs can be computed explicitly as functions of $F_{AP}(t)$:

$$\begin{aligned}
IP(t,a) &= \beta F_{AP}(t-a) \\
\overline{IP}(t) &= \int_0^{T_c^{IPN}} \beta F_{AP}(t-a)da = \int_{\max(0,t-T_c^{IPN})}^{t} \beta F_{AP}(s)ds \quad \text{(A6-1)} \\
\overline{IP}_M(t) &= \int_{\max(0,t-T_c^{IPN})}^{\max(0,t-T_c^{IPN}+T_M)} \beta F_{AP}(s)ds \quad \text{(A6-2)} \\
MI(t) &= \frac{\overline{IP}_M(t)}{\overline{IP}(t)} \quad \text{(A6-3)} \\
N(t) &= \int_0^{\max(0,t-T_c^{IPN})} 2\beta F_{AP}(s)ds + \int_0^t (1-\beta)F_{AP}(s)ds. \quad \text{(A6-4)}
\end{aligned}$$

In the stationary (non realistic) case when $F_{AP}(t) = K_{AP}$, equations (A6-3-A6-4) boil down to

$$\begin{aligned}
\overline{IP}(t) &= \beta T_c^{IPN} K_{AP} \\
MI(t) &= \frac{T_M^{IPN}}{T_c^{IPN}} \\
N(t) &= (1+\beta)K_{AP}t - 2\beta T_c^{IPN} K_{AP}.
\end{aligned}$$

Performing a linear regression of experimental values for $(\overline{IP}(t_n), N(t_n)$ would provide estimates for two combinations of parameters, $\beta T_c^{IPN} K_{AP}$ and $(1+\beta)K_{AP}$,leaving the individual parameters T_c^{IPN}, K_{AP} and β unindentifiable, while providing an additional MI dataset would allow one to estimate T_c^{IPN}.

In the general case when $F_{AP}(t)$ depends on time, we have to compute the solutions numerically. Using formulas (A6-3-A6-4) and the values of K_{AP} parameters in Table A6-1, we compute a deterministic reference solution, $\overline{IP}_M(t)$, $MI(t)$ and $N(t)$ (blue curves in Fig. A6-1), and generate 20 noised datasets, in a similar way as done in additional file 4.
The experimental times are $t_n = 10 + n/2$ for $n = 0, \ldots 12$. Each noised dataset

is then used to identify p=(T_C^{IPN}, β, K_{AP}, t_α , t_δ), by minimizing either

$$J_2(p) \quad = \quad \frac{\sum_t |\overline{IP}(t,p) - \overline{IP}^{exp}(t)|^2}{w_{IP}^2} + \frac{\sum_t |N(t,p) - N^{exp}(t)|^2}{w_N^2}$$

or

$$J_3(p) \quad = \quad \frac{\sum_t |\overline{IP}(t,p) - \overline{IP}^{exp}(t)|^2}{w_{IP}^2} + \frac{\sum_t |N(t,p) - N^{exp}(t)|^2}{w_N^2} + \frac{\sum_t |MI(t,p) - MI^{exp}(t)|^2}{w_{MI}^2}.$$

Given the difference in the range of MI values with respect to that of $\overline{IP}$ and N, the variance of the added noise, distributed according to $\mathcal{N}(0, 5\sigma_X)$, are determined jointly with the optimization weights

$$\begin{aligned} w_X &= \sum_t X^{exp}(t), \quad \text{for } X = \overline{IP},\ N,\ MI \\ \sigma_{MI} &= \frac{w_{MI}}{w_{IP}}, \quad \sigma_N = \frac{w_N}{w_{IP}}, \quad \sigma_{IP} = 1 \end{aligned}$$

Here we can use a simplified optimization criterion compared to criterion (18), by adjusting the noise level to take into account the amplitude of the experimental datasets.An instance of a noised dataset (orange dots) is displayed in Fig. A6-1. When minimizing $J_2(p)$ (red lines),$F_{AP}(t)$ is well recovered, yet both T_c^{IPN} and β are over-evaluated. As a result, the numbers of IP (red lines) are close to the reference ones (in blue), while both the MI, and, to a lower extent, the numbers of neurons are under-evaluated. Minimizing $J_3(p)$ (magenta lines) leads to a better balance between F_{AP}, T_C^{IPN},and β, which improves the fit of MI.

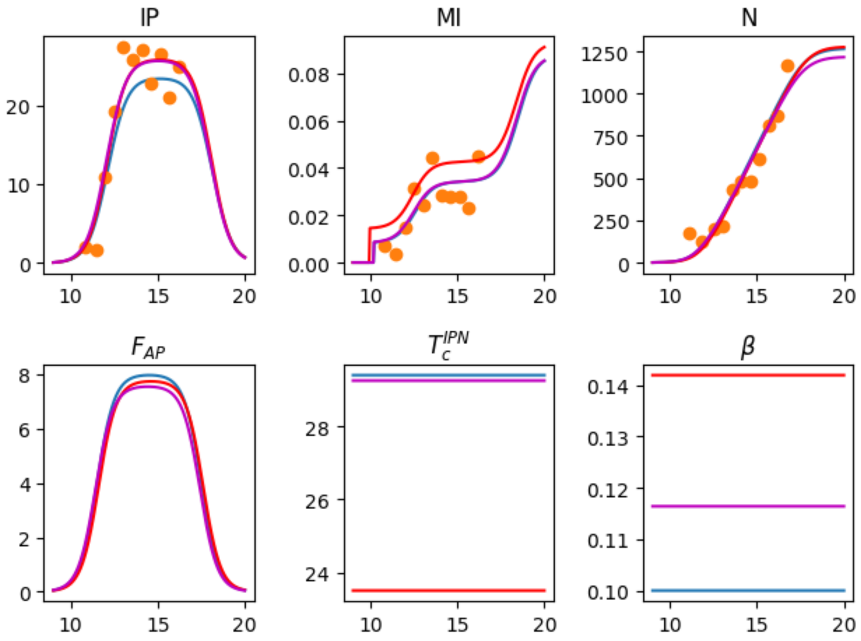

Figure A6-1: Calibrations of $F_{AP}(t)$, β and T_C^{IPN} on a single noised dataset (orange dots), obtained from the reference dataset (blue line). Red lines correspond to the mimimization of $J_2(p)$ (with only cell number datasets); magenta lines to the mimimization of $J_3(p)$ (including and additional MI dataset).

The statistics for each optimized parameter ($\bar{p}$, the mean value, and d_p, the mean absolute deviation from $\bar{p}$) are displayed in Table A6-1. As expected, the gain obtained with $J_3(p)$ mostly impacts the assessment of the cell cycle duration, which is estimated more accurately with respect to the reference value $T_c^{IPN} = 29.4$h (30.2 h, $\sigma = 1.83$) than with $J_2(p)$ (33.19 hours and $\sigma = 6.96$).

Statistics on the parameters optimized from 40 synthetic datasets

	p	T_C^{IPN}	β	K_{AP}	t_-	s_+	t_+	s_-
True		29.4	0.1	8	11.5	2	16.5	2
argmin J_2	$< p >$	33.19	0.099	7.79	11.33		17.46	
	σ_p	6.96	0.027	0.97	0.29		2.	
	$\frac{<p>-p}{p}$	0.13	-0.008	-0.026	-0.015		0.058	
argmin J_3	$< p >$	30.2	0.104	7.99	11.39		16.28	
	σ_p	1.83	0.016	1.16	0.26		0.92	
	$\frac{<p>-p}{p}$	0.027	0.037	-0.001	-0.009		-0.013	

Table A6-1: First row : nominal values of the parameters (in addition, the duration of mitosis is set to 0.5h and parameters s_+ and s_- are excluded from the optimization procedure). Following rows: for each criterion $J_2(p)$ or $J_3(p)$, and each parameter, $< p >$ is the mean value, σ_p, the standard deviation, and $\frac{<p>-p}{p}$ the mean deviation from the mean value.
