# Supplementary material for: A multiscale mathematical model of cell dynamics during neurogenesis in the mouse cerebral cortex
Source: BMC Bioinformatics. 2019 Sep 14;20:470. doi: 10.1186/s12859-019-3018-8 (PMC6744691; doi:10.1186/s12859-019-3018-8)
Supplement: Supplementary file 7 — Identifiability. (PDF 240 kb) [file 12859_2019_3018_MOESM7_ESM.pdf]

Additional file 7. Structural identifiability

In this paragraph we give insight into how the knowledge on the outputs $N(t)$, $\overline{IPP}(t)$ and $\overline{IPN(t)}$ (or alternatively on $N(t)$, $\overline{IPP}(t)$ and $\overline{IP(t)} = \overline{IPP(t)} + \overline{IPN(t)}$) on a given time interval $[0, T]$, enables one to uniquely identify the functions $\gamma(t)$, $\beta(t)$ and $F_{AP}(t)$ on the same time interval. This is formulated mathematically by an injectivity criterion on the map

$$\begin{aligned} \mathcal{A} : C^0([0,T];\mathbb{R})^3 &\longrightarrow C^0([0,T];\mathbb{R})^3 \\ (\gamma, \beta, F_{AP}) &\longrightarrow (\overline{IPP}, \overline{IPN}, N) \end{aligned} \tag{A7-1}$$

To study $\mathcal{A}$, we solve the PDE model with the method of characteristics. Plugging (22) and (23) into (26) we obtain the expression of the model outputs

$$\overline{IPP}(t) = \int_{\max(0,t-T_C^{IPP})}^{t} \gamma(s)\beta(s)F_{AP}(s)ds \tag{A7-2}$$

$$\overline{IPN}(t) = \int_{\max(0,t-T_C^{IPN})}^{t} (1-\gamma(s))\beta(s)F_{AP}(s)ds \tag{A7-3}$$

$$+ \int_{\max(0,t-T_C^{IPP}-T_C^{IPN})}^{\max(0,t-T_C^{IPP})} 2\gamma(s)\beta(s)F_{AP}(s)ds \tag{A7-4}$$

$$\begin{aligned} N(t) &= \int_0^t \left((1-\beta(s))F_{AP}(s) + 2IPN(s, T_C^{IPN})\right) ds \\ &= \begin{cases} \int_0^t (1-\beta(s))F_{AP}(s)ds & \forall t < T_C^{IPN} \\ \int_0^t (1-\beta(s))F_{AP}(s)ds \\ \quad +2\int_0^{t-T_C^{IPN}} (1-\gamma(s))\beta(s)F_{AP}(s)ds & \forall T_C^{IPN} \le t < T_C^{IPN} + T_C^{IPP} \\ \int_0^t (1-\beta(s))F_{AP}(s)ds \\ \quad +2\int_0^{t-T_C^{IPN}} (1-\gamma(s))\beta(s)F_{AP}(s)ds \\ \quad +4\int_0^{t-T_{IPN}-T_{IPP}} \gamma(s)\beta(s)F_{AP}(s)ds & \forall T_C^{IPN} + T_C^{IPP} \le t \end{cases} \end{aligned} \tag{A7-5}$$

where we take into account that $T_{IPN} < T_{IPP}$. In the simple case of constants coefficients $\gamma(t) = \bar{\gamma}$, $\beta(t) = \bar{\beta}$, and $F_{AP}(t) = \bar{F}$, the previous expressions boil down

to,

$$\overline{IPP}(t) = \begin{cases} t\bar{\gamma}\bar{\beta}\bar{F} & \forall\ t < T_C^{IPP} \\ T_C^{IPP}\bar{\gamma}\bar{\beta}\bar{F} & \forall\ T_C^{IPP} \le t \end{cases} \quad \text{(A7-6)}$$

$$\overline{IPN}(t) = \begin{cases} t(1-\bar{\gamma})\bar{\beta}\bar{F} & \forall\ t < T_C^{IPN} \\ T_C^{IPN}(1-\bar{\gamma})\bar{\beta}\bar{F} & \forall\ T_C^{IPN} \le t < T_C^{IPP} \\ \big(T_C^{IPN}(1-\bar{\gamma}) & \\ \quad +2\bar{\gamma}(t-T_C^{IPP})\big)\,\bar{\beta}\bar{F} & \forall\ T_C^{IPP} \le t < T_C^{IPP} + T_C^{IPN} \\ T_C^{IPN}(1+\bar{\gamma})\bar{\beta}\bar{F} & \forall\ T_C^{IPP} + T_C^{IPN} \le t \end{cases} \quad \text{(A7-7)}$$

$$N(t) = \begin{cases} t(1-\bar{\beta})\bar{F} & \forall\ t < T_C^{IPN} \\ t(1-\bar{\beta})\bar{F} & \\ \quad +2(t-T_C^{IPN})(1-\bar{\gamma})\bar{\beta}\bar{F} & \forall\ T_C^{IPN} \le t < T_C^{IPN} + T_C^{IPP} \\ t(1-\bar{\beta})\bar{F} & \\ \quad +2(t-T_C^{IPN})(1-\bar{\gamma})\bar{\beta}\bar{F} & \\ \quad +4(t-T_C^{IPN}-T_C^{IPP})\bar{\gamma}\bar{\beta}\bar{F} & \forall T_C^{IPN} + T_C^{IPP} \le t \end{cases} \quad \text{(A7-8)}$$

The constants T_C^{IPP} and T_C^{IPN} being given and non zero, suppose that two sets of coefficients $(\bar{F}_1,\ \bar{\gamma}_1,\ \bar{\beta}_1)$ and $(\bar{F}_2,\ \bar{\gamma}_2,\ \bar{\beta}_2)$ lead to the same outputs $(\overline{IPP}(t)$, $\overline{IPN}(t)$, $N(t))$. We further assume that $\beta \neq 0$ (at least some neurogenesis involving IP happens). We obtain for $t \geq T_{IPP} + T_{IPN}$

$$\begin{aligned} \overline{IPP}(t) &= T_{IPP}\bar{\gamma}_1\bar{\beta}_1\bar{F}_1 = T_{IPP}\bar{\gamma}_2\bar{\beta}_2\bar{F}_2, \\ \overline{IPN}(t) &= T_{IPN}(1+\bar{\gamma}_1)\bar{\beta}_1\bar{F}_1 = T_{IPN}(1+\bar{\gamma}_2)\bar{\beta}_2\bar{F}_2. \end{aligned}$$

from which we get

$$\bar{\gamma}_1\bar{\beta}_1\bar{F}_1 = \bar{\gamma}_2\bar{\beta}_2\bar{F}_2 \quad \text{(A7-9)}$$

$$\bar{\beta}_1\bar{F}_1 = \bar{\beta}_2\bar{F}_2 \quad \text{(A7-10)}$$

From the assumption $\beta \neq 0$ we obtain from either (A7-9) or (A7-10) that $\bar{\beta}_1\bar{F}_1 = \bar{\beta}_2\bar{F}_2 \neq 0$ and (A7-9) leads to $\bar{\gamma}_1 = \bar{\gamma}_2$.
We then use (A7-8) for $t \leq T_{IPN}$

$$t(1-\bar{\beta}_1)\bar{F}_1 = t(1-\bar{\beta}_2)\bar{F}_2$$

which leads to $\bar{\beta}_1 = \bar{\beta}_2$.
We have therefore proved the identifiability of the model for constant parameters : if two sets of parameters $(\bar{\gamma}^j, \bar{\beta}^j, \bar{F}^j)$ for $j = 1, 2$ lead to the same outputs (A7-6-A7-8) then $\bar{\gamma}^1 = \bar{\gamma}^2$, $\bar{\beta}^1 = \bar{\beta}^2$ and $\bar{F}^1 = \bar{F}^2$.
in the general case of time varying coefficients γ, β and F_{AP}. We reformulate the model in terms of the input flux $F_{AP}(t)$, the IP flux F_I and the IPP flux F_P

$$F_I(t) = \beta(t)F_{AP}(t) \quad \text{(A7-11)}$$

$$F_P(t) = \gamma(t)\beta(t)F_{AP}(t) \quad \text{(A7-12)}$$

Expressions (A7-2-A7-5) of the model outputs become

$$\overline{IPP}(t) = \int_{\max(0,t-T_{IPP})}^{t} F_P(s)ds, \tag{A7-13}$$

$$\begin{aligned}\overline{IPN}(t) &= \int_{\max(0,t-T_{IPN})}^{t} (F_I(s) - F_P(s))ds \\ &\quad + \int_{\max(0,t-T_{IPP}-T_{IPN})}^{\max(0,t-T_{IPP})} 2F_P(s)ds,\end{aligned} \tag{A7-14}$$

$$N(t) = \begin{cases} \int_0^t (F_{AP}(s) - F_I(s))ds, & \forall t < T_{IPN}, \\ \int_0^t (F_{AP}(s) - F_I(s))ds \\ \quad +2\int_0^{t-T_{IPN}} (F_I(s) - F_P(s))ds, & \forall T_{IPN} \leq t < T_{IPN} + T_{IPP}, \\ \int_0^t (F_{AP}(s) - F_I(s))ds \\ \quad +2\int_0^{t-T_{IPN}} (F_I(s) - F_P(s))ds \\ \quad +4\int_0^{t-T_{IPN}-T_{IPP}} F_P(s)ds, & \forall t \geq T_{IPN} + T_{IPP}. \end{cases} \tag{A7-15}$$

Suppose that two sets of fluxes $(F_I^j(t), F_P^j(t), F_{AP}^j(t))$ produce the same outputs $(\overline{IPP}(t), \overline{IPN}(t), N(t))$ for all $t \geq 0$. We show that it implies

$$F_I^j(t) = F_I(t), \quad F_P^j = F_P(t), \quad F_{AP}^j(t) = F_{AP}(t), \quad j = 1, 2, \text{ for all } t \tag{A7-16}$$

For sake of simplicity we only sketch the proof in the case $T_{IPP} = T_{IPN} = T$ which simplifies Equations (A7-13-A7-15).

- For $0 \leq t \leq T$ we obtain

$$\begin{aligned}\overline{IPP}(t) &= \int_0^t F_P^j(s)ds, \\ \overline{IPN}(t) &= \int_0^t (F_I^j(s) - F_P^j(s))ds, \\ N(t) &= \int_0^t (F_{AP}^j(s) - F_I^j(s))ds,\end{aligned}$$

  which we derive in t in order to obtain (A7-16) for $0 \leq t \leq T$.
- For $T \leq t \leq 2T$ equations (A7-13-A7-15) become

$$\begin{aligned}\overline{IPP}(t) &= \int_{t-T}^t F_P^j(s)ds, \\ \overline{IPN}(t) &= \int_{t-T}^t (F_I^j(s) - F_P^j(s))ds + 2\int_0^{t-T} F_P^j(s)ds, \\ N(t) &= \int_0^t (F_{AP}^j(s) - F_I^j(s))ds + 2\int_0^{t-T} (F_I^j(s) - F_P^j(s))ds,\end{aligned}$$

  Deriving the first equation in t provides $F_P^1(t) - F_P^1(t-T) = F_P^2(t) - F_P^2(t-T)$ and using the identity (A7-16) already obtained on $[0, T]$ we get $F_P^1(t) = F_P^2(t)$

on $[T, 2T]$ as well. Then from the second equation we obtain $F_I^1(t) - F_I^1(t-T) = F_I^2(t) - F_I^2(t-T)$ from which we get $F_I^1(t) = F_I^2(t)$ on $[T, 2T]$. Eventually the third equation provides $F_{AP}^1(t) = F_{AP}^2(t)$ on $[T, 2T]$ since all the other terms have already been proved equal.

- A similar argument leads to (A7-16) for $t > 2T$.

From the identifiability of the fluxes (A7-16) can we conclude to the identifiability of γ, β and F_{AP} ? Clearly, since $F_{AP}^1 = F_{AP}^2$, if this quantity is non zero then $F_I^1 = F_I^2$ leads to $\beta^1 = \beta^2$ (these three equalities hold for all t). We then obtain $\gamma^1 = \gamma^2$ from $F_P^1 = F_P^2$.
In practice however, recovering some estimates for F_I, F_P and F_{AP} from the data is feasible but it does not ensure that the parameters $\gamma(t)$ and $\beta(t)$ would satisfy the constraints arising from there biological meaning (namely that they should be within $[0, 1]$).

Note that this result -in both the constant and varying cases- highlights the crucial importance of having access to the IPP dataset, on top of the IP and N datasets, in the identifiability -and therefore the calibration- of the model.
It remains however rather theoretical. In practice we only have access to experimental data within a limited and discretized time window. Rather than address the inversion problem of map $\mathcal{A}$ we have chosen to set $\beta(t) = 1$, and to look for γ and F_{AP} in the finite dimension functional spaces described by (15) and (16).
